# Seasonality of *Plasmodium falciparum* transmission: a systematic review

**DOI:** 10.1186/s12936-015-0849-2

**Published:** 2015-09-15

**Authors:** Robert C. Reiner, Matthew Geary, Peter M. Atkinson, David L. Smith, Peter W. Gething

**Affiliations:** Fogarty International Center, National Institutes of Health, Bethesda, MD USA; Department of Epidemiology and Biostatistics, Indiana University School of Public Health, Bloomington, IN USA; Department of Entomology, University of California, Davis, CA USA; Department of Biological Sciences, University of Chester, Chester, UK; Faculty of Science and Technology, Engineering Building, Lancaster University, Lancaster, LA1 4YR UK; Faculty of Geosciences, University of Utrecht, Heidelberglaan 2, 3584 CS Utrecht, The Netherlands; School of Geography, Archaeology and Palaeoecology, Queen’s University Belfast, Belfast, BT7 1NN Northern Ireland, UK; Geography and Environment, University of Southampton, Highfield, Southampton, SO17 1BJ UK; Center for Disease Dynamics, Economics and Policy, Washington, DC, USA; Spatial Ecology and Epidemiology Group, Department of Zoology, University of Oxford, Oxford, UK

**Keywords:** *Plasmodium falciparum*, Seasonality, Climatic drivers

## Abstract

**Background:**

Although *Plasmodium falciparum* transmission frequently exhibits seasonal patterns, the drivers of malaria seasonality are often unclear. Given the massive variation in the landscape upon which transmission acts, intra-annual fluctuations are likely influenced by different factors in different settings. Further, the presence of potentially substantial inter-annual variation can mask seasonal patterns; it may be that a location has “strongly seasonal” transmission and yet no single season ever matches the mean, or synoptic, curve. Accurate accounting of seasonality can inform efficient malaria control and treatment strategies. In spite of the demonstrable importance of accurately capturing the seasonality of malaria, data required to describe these patterns is not universally accessible and as such localized and regional efforts at quantifying malaria seasonality are disjointed and not easily generalized.

**Methods:**

The purpose of this review was to audit the literature on seasonality of *P. falciparum* and quantitatively summarize the collective findings. Six search terms were selected to systematically compile a list of papers relevant to the seasonality of *P. falciparum* transmission, and a questionnaire was developed to catalogue the manuscripts.

**Results and discussion:**

152 manuscripts were identified as relating to the seasonality of malaria transmission, deaths due to malaria or the population dynamics of mosquito vectors of malaria. Among these, there were 126 statistical analyses and 31 mechanistic analyses (some manuscripts did both).

**Discussion:**

Identified relationships between temporal patterns in malaria and climatological drivers of malaria varied greatly across the globe, with different drivers appearing important in different locations. Although commonly studied drivers of malaria such as temperature and rainfall were often found to significantly influence transmission, the lags between a weather event and a resulting change in malaria transmission also varied greatly by location.

**Conclusions:**

The contradicting results of studies using similar data and modelling approaches from similar locations as well as the confounding nature of climatological covariates underlines the importance of a multi-faceted modelling approach that attempts to capture seasonal patterns at both small and large spatial scales.

**Electronic supplementary material:**

The online version of this article (doi:10.1186/s12936-015-0849-2) contains supplementary material, which is available to authorized users.

## Background

Like many infectious diseases, malaria incidence often displays seasonal variation. The nature and extent of the seasonality varies enormously from place-to-place and from year-to-year. Temporal variation in malaria transmission is, along with its spatial distribution, among the most basic aspects of its epidemiology. Knowledge of the main drivers of seasonality, their timing, and interaction with malaria transmission in a given location can facilitate effective planning and implementation of routine control and treatment activities. Some interventions can be more effective if deployed at seasonally optimal times. Seasonal malaria chemoprevention, for example, which involves the preventative administration of anti-malarial drugs to young children [1], is optimally targeted at regions with a short, intense, malaria transmission season, and requires accurate timing within that season [2]. An understanding of seasonality is also important when measuring and describing geographical patterns of malaria risk [3] and burden: observations made at different months in the year are difficult to compare without reference to a known underlying seasonal signal. Similarly, seasonality affects interpretation between different types of malaria data: the overall and age-specific relationships between vector population density, the entomological inoculation rate (EIR), the force of infection, infection prevalence or parasite rate (PR), disease incidence and mortality all respond differently and non-linearly in areas of differing seasonality [4, 5]. Further, adequate models of seasonality of malaria would be useful for understanding changing risks for travellers.

Despite the clear importance of quantifying the seasonality of malaria, data describing it are not widely available. While those involved in day-to-day disease control and treatment may harbour detailed knowledge of local seasonal patterns, there remains no single resource providing consistent and comparable data on the extent, timing, and determinants of seasonality at regional-to-global scales. The first challenge is one of definition. In a malaria context, the term seasonality encapsulates a complex and multi-faceted phenomenon which remains inconsistently defined, described, and interpreted. A basic description of seasonality in a location would include the relative magnitude, timing of onset, and duration of different seasons. These attributes must be characterized separately for each malaria metric of interest. Crucially, characterization of the “typical” seasonal pattern is likely to differ from that observed in any single year, since inter-annual variation is often substantial. Malaria seasons often start earlier or end later, last for a longer or shorter duration, or are more or less pronounced from one year to the next, and so this year-to-year variation around an average pattern must be captured and described.

A second challenge, leading directly from the first, is the availability of standardized and geolocated data describing patterns of seasonality that can be compared across a wide set of locations. While there is a degree of consensus on the broad global patterns of seasonality, this falls considerably short of a geographically detailed, quantitatively rich characterization that could support in-depth control planning. An understanding of the geographical distribution of malaria has benefited enormously from the proliferation of standardized [6], often nationally representative [7, 8] cross-sectional parasite rate surveys, and their assimilation into geospatial models [3, 9], but such data are not well suited to capturing seasonal variation. Conversely, longitudinal or other time-series data that are ideal for analysing temporal patterns are less commonly obtained, address a disparate set of malaria metrics, tend to be unevenly distributed geographically [10] and can be prone to biases and missing data [11].

This scarcity of robust and comparable data means the empirical evidence based on patterns of seasonality remains unconsolidated. The purpose of this review is to audit the extant literature on seasonality of *Plasmodium falciparum*, and to provide a quantitative summary in terms of: (1) the geographical regions represented; (2) the type of malaria metrics measured; (3) the climatic drivers identified; and (4) the analytical approach taken to explore seasonal dynamics, which include a broad class of both statistical and mechanistic modelling approaches.

## Methods

### Constructing a systematic bibliographic database

**Fig. 1:**
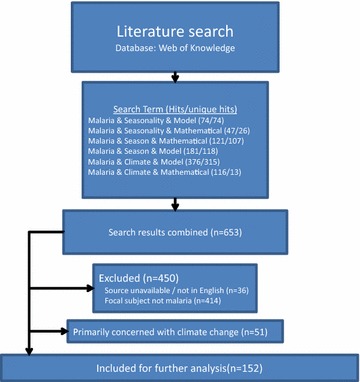
Flow chart: Summary of systematic search. Number of papers returned by each of the six search terms selected to systematically compile a list of papers, from the academic search engine Web of Knowledge, relevant to the seasonality of *Plasmodium falciparum* transmission

The intended scope of this review was all studies in the scientific literature that have either explicitly or implicitly observed, described or modelled malaria seasonality and its drivers. Hundreds of such studies exist from sites around the world, fostered in part by the increasing diversity and availability of environmental and climatic covariates arising from both satellite-sensor imagery and improved on-the-ground data collection techniques. Six search terms were selected to systematically compile a list of papers relevant to the seasonality of *P. falciparum* transmission. These terms were then entered into the academic search engine Web of Knowledge [12] and new papers from each search term added to the list each time (Fig. 1).

These search terms were deliberately broader than the scope of this review to capture as many potentially relevant papers as possible, with the large set of returned studies then successively screened for inclusion according to a set of criteria described below. It must be noted that locations where malaria transmission is relatively non-seasonal would be less likely to be identified through this criteria (e.g., Papua New Guinea) and as such the final set of papers do not represent where malaria exists, but rather where it may be seasonal. The abstract and titles of each paper were checked to identify papers with a focal subject that was not malaria seasonality. These papers were removed from consideration at this stage (450 papers). To systematically quantify the remaining broad assembly of literature, a classification questionnaire was designed and then applied to every publication. The ‘questionnaire’ was structured as follows:

1. Does the paper try to understand malaria seasonality or produce a model of the relationship between malaria and environmental variables?
2. Does the paper include environmental or climatic variables and, if so, which variables are considered?
3. Are the data used by the authors new and if so what type of data is used to represent malaria?
4. In which locations is the study based?
5. What time periods does the paper cover?
6. What was the time scale of the data?
7. Is the analysis primarily mechanistic or statistical in nature and what are the main methods?
8. What aspects of seasonality does the paper consider (e.g. timing of malaria peaks, difference between minimum and maximum, environmental drivers)?
9. Is the paper primarily concerned with climate change?
10. Is the method of particular interest because of its novelty or because it creates a solution to a particular problem?
11. Does the paper call for work on this issue?

The answers to these questions were recorded by one of the two lead authors to produce a reference for the comparison of approaches to investigating malaria seasonality as well as the global coverage of these attempts. Due to the straightforward nature of the questions (and based on complete agreement for an initial subset of studies), each manuscript was only categorized by one author. There were instances where the same data set was analysed in two different ways within two different manuscripts. In these cases, each analysis was counted separately.

## Results

Classifying each manuscript using the above questionnaire generated a considerable amount of detailed information. For brevity, this information is summarised in general terms below. To provide readers with increasing levels of detail, twelve supplemental tables are included in Additional files 1, 2, 3, 4, 5, 6, 7, 8, 9, 10, 11, 12, 13 and the raw database is provided as Additional file 14, detailed manuscript-specific review of the incorporation of the main climatological drivers is included in Additional file 15. In total, 152 manuscripts were identified that satisfied the criteria for inclusion (Flowchart 1, Additional file 16). Although these manuscripts considered somewhat similar questions, many answered them with significantly different data and approaches. In what follows, common patterns are identified within the analyses but it is important to caution against lumping all the analyses together and identifying a single result. It is inappropriate to simply average results derived from yearly data on the country scale with monthly data on the county scale with weekly data on the city scale even if they all analyze rainfall’s influence on malaria incidence. Although the large number of studies should provide information that can be directly synthesizable to direct public policy, the lack of consistent data across studies—either in terms of common definitions of metrics or drivers or in terms of consistent spatial and temporal scales—renders a more synthetic analysis of the results of this review inappropriate. Further, without the original data, it is not possible to assess which of the broad class of modelling approaches used was most appropriate for each analyses.

### Regions

**Fig. 2:**
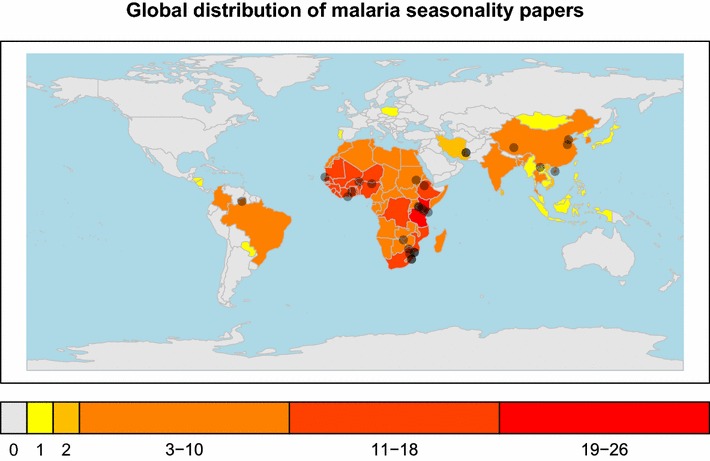
Global distribution of malaria seasonality studies. The frequency with which countries are the focus of malaria seasonality studies is plotted. Studies that considered individual locations are indicated by grey points on the map

Across the 152 manuscripts, 130 defined a location or region of interest. Although most of these manuscripts focused on a single study location or country, five manuscripts separately analysed seasonality of multiple locations or countries, while fourteen manuscripts lumped multiple countries together into a single analysis. Throughout what follows, because conclusions within a single paper were often different for different study sites, every study location was considered independently and each location site-by-manuscript combination is referred to as a ‘study.’ In total, there were 159 studies. Across these studies, the vast majority (74.2 %, 118/159) concerned the effects of climate and seasonality on malaria in Africa (see Fig. 2). Five studies covered all of Africa, while nine focused on regions of Africa (Additional file 1). Excluding these regional and continent-wide studies, there were 104 studies of 26 African countries. Outside Africa, there were 28 studies within Asia, with China (eight) and India (four) being the two most studied countries (Fig. 2). Beyond these locations, there were eleven studies in South and Central America, two studies in Iran and two studies in Europe (one each in Portugal and Poland). For a complete classification of the frequency of location utilization, see Additional file 1.

### Malaria metrics

Malaria transmission has been evaluated historically using various metrics. Abundances or frequency of blood feeding by anopheline mosquitoes, the vectors of malaria, have been used as a proxy for transmission, and a measure of transmission potential. EIR, which is the product of the number of vectors attempting to feed and the percent of mosquitoes actively infective, gives quantitative estimates of the number of infective bites per person per unit time. Prevalence of infections or incidence of clinical cases, detected actively in the community or passively at health facilities, respectively, provide more direct measures of the current level of transmission and disease within human hosts. Such metrics of malaria are representative of different aggregated temporal windows of transmission, which complicates attempts to link environmental drivers and malariometric outcomes of seasonal transmission. In what follows, only the studies that analysed malaria within a specific location, country or region will be addressed.

Across the 159 studies, 29 used mosquito abundance as a malaria metric (Additional file 17a). The majority of these studies concerned regions of Africa. Incidence of clinical disease was the most frequently investigated malaria metric (73 studies), and most of the regions of the globe with malaria were represented by studies using this metric (Additional file 17b). EIR and infection prevalence were only investigated in regions of Africa (Additional file 17b, c, respectively). As with mosquito abundance, EIR and prevalence were far less frequently studied relative to incidence (six and 22 studies, respectively). Beyond these four metrics, there were a variety of alternative metrics (e.g., malaria related death) that were used infrequently and aggregated into an ‘other malaria metric’ group.

### Climatic drivers

The most commonly reported aspect of malaria seasonality was observed temporal relationships between a given malaria metric and a given putative environmental or climatic driver of seasonality. The most direct method of obtaining data in specific locations is to take in situ measurements of variables of interest or, for climatic drivers, to use local weather stations. However, accurate and complete records of all variables of interest across space and time may be lacking, particularly in many of the resource-poor locations of interest for malaria transmission. Over wider areas the use of nationally collected data from weather station networks may be more appropriate (e.g. National Meteorological Services Agency in Ethiopia, Islamic Republic of Iran Meteorological Organisation, and China Meteorological Administration). A common source of global climatic data is WORLDCLIM which, by interpolating data to cover areas away from initial weather station locations, has made available fine resolution interpolated surfaces from several trusted weather databases over a 50 year time period [13]. An alternative to terrestrial weather and climate data is provided by satellite sensors such as Moderate-resolution Imaging Spectroradiometer (MODIS) on the Terra and Aqua satellites; [14]. Unlike data from weather stations which can be patchy in their coverage, satellite sensors can achieve complete global coverage, and data from satellite-mounted sensors such as the Advanced Very High Resolution Radiometer can be used to infer variables such as sea surface temperature [15], water vapour levels [14], atmospheric gas concentrations [16] and precipitation [17] as well as compute vegetation indices such as the normalized difference vegetation index (NDVI) which measures the “greenness” of vegetation based on its reflectance. The choice of data source on climatic drivers of malaria metrics will depend on various factors such as the spatial and temporal resolutions, time period and location of the study in question.

**Fig. 3:**
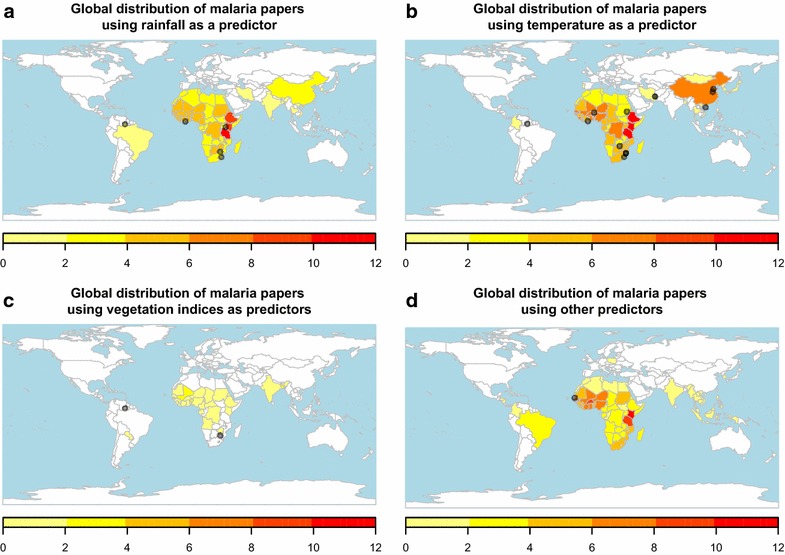
Distribution of malaria seasonality studies by climatological driver. The frequency that climatological covariates are identified as significant drivers of malarial metrics is plotted for rainfall (**a**), temperature (**b**), vegetation indices (**c**) and all other climatological covariates (**d**). Studies that considered individual locations are indicated by grey points on the maps. Note that several studies used no climatological drivers in their analysis and are thus not included on any panel in this figure. Each interval is left-closed and right-open except for the final interval

The majority of studies analysed the relationship between malaria metrics and temperature or rainfall (40.3 %, 64/159 and 34 %, 54/159, respectively; Fig. 3a, b). Satellite-derived indices quantifying vegetation coverage were also frequently investigated (11.3 %, 18/159; Fig. 3c), often in conjunction with temperature and/or rainfall. All other potential drivers (e.g., relative humidity, wind speed and direction, sunspots) were either used rarely (2.5 %, 4/159; Fig. 3d) or in conjunction with a subset of the three main drivers (12.6 %, 20/159). Although it would also be informative to know which variables were tested for but not found to be significant drivers of malaria, a large proportion of studies omitted this information which led us to not summarize those results from the few studies that included negative results. Here, findings from those studies that used statistical methods to investigate seasonal drivers is summarised.

#### Temperature

**Fig. 4:**
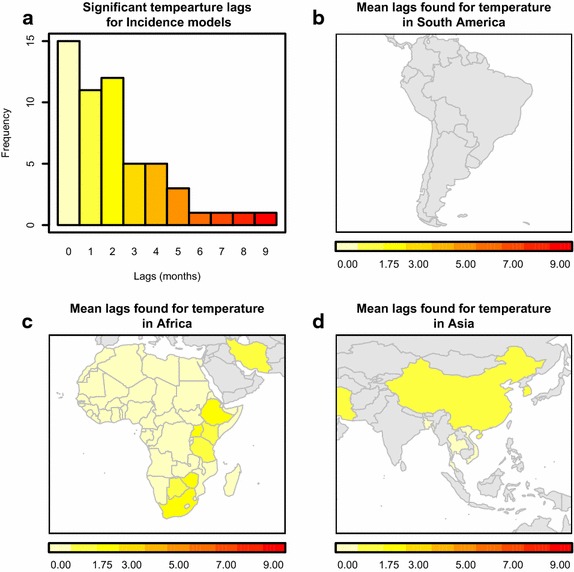
Reported relationships between temperature and malaria incidence. In ** a** the distribution of all significant temperature lags to incidence is plotted. Different approaches used different forms of monthly temperature in their model. In ** b**–**d** only the mean significant temperature lag is plotted by country in South America, Africa and Asia respectively

Across studies that used statistical models, temperature covariates were found to be a significant driver of malaria seasonality more frequently than any other climatological drivers (64 studies). Amongst temperature-based variables, minimum monthly temperature was most frequently found to have a significant relationship with temporal malaria metrics (24 studies), followed by maximum monthly temperature (19 studies) and mean monthly temperature (12 studies). The range of significant time lags between monthly temperature and malaria metrics varied by both region and, as expected, malaria metric. As with all studies, the dominance of malaria incidence-based investigations within the literature was again clear. However, given the history of laboratory and field-based experiments correlating temperature with mosquito population dynamics [18], it is not surprising that 20 studies found a significant relationship between some measure of monthly temperature and vector abundance (Additional file 2). All but one of these was a zero-month lag, with a single study lagging temperature by two months and all but one of the studies concerned regions in Africa (one was in Portugal). Incidence was the most frequently investigated malaria metric, and of the 73 statistical analyses that correlated climatological drivers with incidence, 36 found a significant relationship between monthly temperature and incidence. Temperature was a significant driver in incidence studies throughout the Old World, with lags ranging from zero to nine months (Fig. 4). EIR, the other direct measure of current transmission activity within a region, was found to be significantly related to temperature in four studies, at lags from zero to one months, all within Africa (Additional file 1). Finally, across the four studies that found significant relationships between monthly temperature and prevalence, all again occurred in Africa and most found a maximum lag of two months to be significant (Additional file 1). A more detailed break-down of the number of times a specific temperature variable was found to be a significant driver of a specific malaria metric in a specific region can be found in Additional files 1, 2, 3, 4, 5, 6, 7, 8, 9, 10, 11, 12, 13.

#### Rainfall

**Fig. 5:**
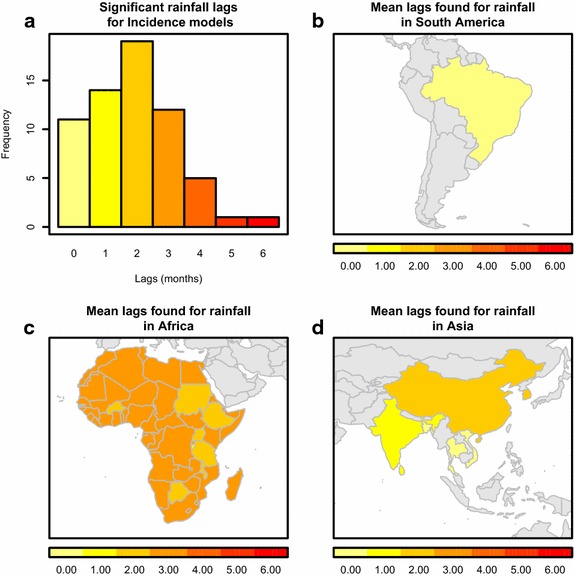
Reported relationships between rainfall and malaria incidence. In ** a**, the distribution of all significant rainfall lags to incidence is plotted. Different approaches used different forms of monthly rainfall in their model. In ** b**–**d**, only the mean significant rainfall lag is plotted by country in South America, Africa and Asia respectively

54 studies across the globe found rainfall to be a significant predictor of malaria seasonality. Ten studies found a significant relationship between mean monthly rainfall and malaria metrics. Presumably driven by the non-linear relationship between rainfall and malaria, many investigators assessed specific statistics of rainfall other than mean monthly amount, such as seasonal rainfall [19], total rainfall during a set period [20], and various other indices of variation. Four studies found a significant relationship between rainfall and vector abundance (Additional file 2) with lagged relationships between one and two months. For both incidence and EIR, lags ranged from zero to six months (37 studies, Fig. 5; two studies, Additional file 2). Across the four studies that found significant relationships between monthly rainfall and prevalence, all found a zero month lag to be statistically significant (Additional file 2). A more detailed regional break-down of the number of times a specific rainfall variable was found to be a significant driver of a specific malaria metric can be found in Additional files 1, 2, 3, 4, 5, 6, 7, 8, 9, 10, 11, 12, 13.

#### Vegetation indices

18 studies found a satellite-derived vegetation index to be a significant driver of malaria metrics; all but three used NDVI. Across various monthly vegetation indices, four studies found a significant correlation with vector abundance (Additional file 2). All of these were zero month lags and located in either Africa or Asia. Significant relationships between vegetation indices and incidence were found across the globe at zero to three month lags (nine studies, Additional file 2). Two studies found significant concurrent relationships between vegetation indices and EIR in Africa (Additional files 1, 2) and across the three studies that found significant relationships between monthly vegetation indices and prevalence, also all in Africa, lags of zero and one month were identified. (Additional files 1, 2). Again, more detailed break-downs of these results are provided in Additional files 1, 2, 3, 4, 5, 6, 7, 8, 9, 10, 11, 12, 13.

### Approaches: statistical methods

The database of seasonality studies included a wide range of statistical modeling approaches used to investigate empirical associations between malaria metrics and environmental drivers (126 studies). These ranged from descriptive approaches to fuzzy logic models and complex spatio-temporal methods. 22 studies used methods classified by the authors as ‘simple’. This included descriptive methods and purely correlative approaches with no model fitting. The largest number of studies, 50, used classes of regression methods including both parametric and non-parametric. Some included residual error structures such as autoregressive terms. Logistic and Poisson regression were common approaches within this group along with several multivariate methods and mixed models. A further 20 studies used spatial methods, including spatial regression and spatial autocorrelation terms, along with geostatistical and niche modelling methods, and three additional studies used explicitly spatio-temporal methods. Twelve of the studies using statistical methods used Bayesian approaches. Of these, three were spatial models and one used spatio-temporal methods.

The overall number of papers published per year increased towards the present (Additional file 18). A clear trend of increasing modelling sophistication was evident, with a proportional decline in papers using simple statistical methods whilst spatial and Bayesian approaches proportionally increased. Almost all of the ‘simple’ studies concentrated on Asia and Africa (21/22 studies, Additional file 9) and more than half were concerned with malaria cases or incidence (twelve studies, Additional file 4). Rainfall and temperature predictors were commonly used within this group of studies (eight studies and six studies respectively; Additional file 6). Among the models using regression methods the most common malaria metrics investigated were again number of cases and incidence (23 studies, Additional file 4). However, within this group the diversity of malaria metrics investigated was greater than for simple approaches (Additional file 4). The majority of studies using regression methods dealt with Africa (38 studies) but there were also examples in Asia (ten studies), the Americas (one study) and Europe (one study). Regression methods, perhaps due to the breadth of studies using these approaches, used the most diverse range of predictor variables. Malaria cases and incidence were again well represented by studies using Bayesian methods (eight studies, Additional file 4). However, the two studies using spatio-temporal Bayesian models investigated environmental drivers of malaria prevalence [21] and vector abundance [22]. Similarly the spatio-temporal regression models were concerned with PR rather than number of cases and incidence [23, 24]. Bayesian modelling approaches were most commonly associated with temperature as a predictor along with rainfall (Additional file 6) in many cases and were mostly focused on Africa (Additional file 9).

### Approaches—mechanistic models

31 studies investigated the possibility of incorporating seasonality, or seasonal drivers, into mechanistic models of malaria response variables. The majority of these studied malaria in Africa, but there were also several investigations in Asia and South America (Additional file 19). From the initial models of Ross and then Macdonald [25], mechanistic models of malaria have, in general, not greatly deviated from the original framework [26]. There have been a few exceptions to this general observation, and some of the most complex mechanistic modeling approaches have also been adapted to incorporate seasonal differences in malaria. As with the statistical models, there are stark differences in the modeling approach between models that attempt to model monthly malaria incidence data or parasite rate surveys and models that attempt to model mosquito abundance. However, as was true for the statistical approaches, local rainfall and temperature were the most frequently used climatological covariates used to drive temporal variation in malaria.

#### Mosquito abundance

Processes affecting *Anopheles* abundance are known to be related in a nonlinear way with temperature [18]. If the ambient temperature is too cold or too hot, vectors of malaria have a diminished probability of survival. Thus, considerable effort has gone into identifying the optimal temperature window for *Anopheles*. Incorporating temperature into a model of the suitable range of mosquitoes (and then further a suitable range of malaria) has resulted in global maps of malaria potential [3]. Additionally, the potential that the regions of the globe that are within the optimal temperature window for *Anopheles* may shift or expand with global climate change has resulted in numerous investigations and publications [27]. Although much of the work has concerned defining the spatial distribution of locations that have suitable temperature for malaria at any point during a year, several efforts have further investigated the seasonality of mosquito abundance and climatic drivers’ effect on abundance.

Martens et al. [28] modelled the death rate of mosquitoes as a function of temperature in Celsius, *g*(*T*), as:

1 $$\begin{aligned} g(T)=\frac{1}{-4.4+1.31T-0.3T^2} \end{aligned}$$

From basic maps of climate suitability [18] to being used as an integral part of complex malaria models [29, 30], this equation/functional form, or an approximation of it, has been used extensively. Other incorporations of temperature to identify climate suitability have either taken a simple approach of directly defining a window outside of which a mosquito population could not be sustained [31] or using a similar but mathematically different functional form such as the logistic equation used by Lourenço et al. [32]. In addition to temperature, functional forms have been used to incorporate other climatological covariates such as rainfall and temperature into estimates of climate suitability for *Anopheles*. As with statistical models of mosquito abundance, there was no estimated lag between the climatological covariates and mosquito abundance.

Complex agent-based models whose primary focus is based on mosquito abundance that incorporate mosquito population ecology and impacts of multiple simultaneous interventions have also been built to accommodate multiple climatological drivers as well as some of their interactions. Eckhoff et al. [33] explicitly tracked cohorts of eggs through their life cycle using mechanistic relationships implemented at the individual level. Modelling local population dynamics (as opposed to well-mixed patches common to mechanistic models defined by differential equations) may allow for locally optimized control strategies once parameterised for a specific location.

#### Malaria incidence

Several mechanistic models included within our review concern primarily the mathematical properties of models that permit intra-annual variation. Chitnis et al. [34] and Dembele et al. [35] both analysed periodically fluctuating parameters within a larger system of differential or difference equations. Chitnis et al. incorporated considerable complexity, especially with respect to the life cycle of *Anopheles*, and both analyze the asymptotic stability of their system as well as investigate the effects of various control efforts. Although these models are not directly applied to data, they provide a rigorous framework within which seasonally fluctuating variables, driven by climate or otherwise, can be incorporated. As noted in a recent review of mechanistic models of mosquito-borne pathogens [26], the complexity of a mechanistic model is typically determined by the exact purpose of the research.

A variety of compartmental models of malaria have incorporated temperature and rainfall to different ends. For example, Massad et al. [36] incorporated both a seasonal sinusoidal driver of mosquito abundance and a second host population into their compartmental modelling approach to assess the risk of travellers to a region with endemic malaria, but in doing so they ignored the incubation period for both host and mosquito. Conversely, Laneri et al. [37] used a single host population, but also incorporated rainfall, incubation periods and secondary infection stages to separate the roles of external forcing and internal feedbacks in inter-annual cycles of transmission.

In general, the vast majority of mechanistic models of malaria incidence that incorporate seasonality or climate are bespoke to address a specific concern. There are, however, several important exceptions. Several research groups have spent the last decade (or more) developing extremely complex and detailed models of malaria. Combining statistical approaches, mechanistic models and in some cases fuzzy logic, these models attempt to recreate transmission patterns at large scales. Amongst these approaches, the utilization of climate and climatic drivers differs. Researchers from Imperial College and the London School of Hygiene & Tropical Medicine built an agent-based simulation model of malaria transmission fitted to 34 transmission settings across Africa [38]. Using seasonal profiles of EIR fitted to different regions they categorized transmission settings into different intensities and identified those locations where reasonable control efforts would have the largest impact. The Liverpool Malaria Model [39] models both malaria and the climatic drivers themselves and incorporates rainfall and temperature to drive the vector population. This complex model has been updated to incorporate further complexities [30] and then calibrated and validated on data from West Africa [40]. Quantities such as the “start” and “end” of the malaria season were simulated and compared well with observed values where applicable. This model, as noted in [40], does not incorporate fine-scale hydrologic variability (extensive data to support its inclusion are lacking). This has been proposed as an explanation as to why year-to-year comparisons between simulations and observations at single locations are generally only weakly correlated.

Bomblies et al. [41] introduced a modelling approach that explicitly incorporates hydrologic variability into vector abundance and then malaria incidence. In direct response to the typical mismatch of scales between the resolution of climatic drivers and the scale of vector population dynamics, the Hydrology, Entomology and Malaria Transmission Simulator (HYDREMATS) uses soil moisture and local hydrology to calibrate a model that captures mosquito abundance at a scale much closer to what is seen in the field, and has been used in several small scale validation and calibration studies [42, 43]. The inclusion of hydrology implicitly incorporates a lag between rainfall and malaria that is non-linearly determined based on ground cover, control practices and size of natural pools within the community. This level of high-resolution hydrological detail is difficult to obtain, or accurately simulate for entire regions or countries.

## Discussion/conclusion

Following an exhaustive literature search, 152 manuscripts that either explicitly or implicitly addressed the seasonality of malaria were categorised. The vast majority of these efforts did not, in fact, attempt to quantify or describe the patterns of seasonality per se, but instead associated malaria data with climatic data. However, because the climatological covariates themselves follow seasonal patterns (some more strongly than others), linking climate with malaria, even at a lag, indicates the potential presence of seasonality. The two clearest aspects of these studies that partitioned the existing literature, somewhat predictably, were the types of data (both explanatory and response) and the types of analyses (generally speaking, statistical versus mechanistic). In every combination, although the limitations of available data soften the conclusions, the presence of variation in ‘seasonality’ seems to be both conditioned and driven by location and climate.

The “seasonality” of malaria within any location is the end result of a series of complex processes. In certain locations, non-linear feedbacks between the multiple stochastic processes (e.g., mosquito population dynamics) may render any individual aspect of seasonality unpredictable. While broadly predictable at coarse scales of time and space, fine-scale variation in climate, combined with fine-scale variation in parasite, mosquito, human and zoonotic density render fine-scale prediction of malaria an extremely difficult task. Considering just mosquitoes, there are approximately 70 mosquitoes of the genus *Anopheles* that can transmit malaria and the dominant species varies greatly across the globe [44]. The ecology of these mosquitoes can vary dramatically, and there can be differences in behaviour within a species across its range; further, the influences of environment can likewise vary considerably. Adding to the complexity of the situation, many locations have multiple important species, and the timing of “peak” transmission can be influenced by the combined population dynamics of these species. Linking specific climatological drivers to malaria transmission, for the purpose of identifying seasonality or merely for prediction of future transmission, must be mediated by the specific ecology of the mosquito species in that location.

As discussed above, the increase in resolution (both spatially and temporally) of satellite-based climatological covariates has greatly contributed to the analyses performed and, in many cases, the amount of variation in malaria explained. Additionally, improved data collection and data maintenance from existing weather stations has provided ground reference data with which the satellite sensor data can be validated. Due to the necessary transmission steps that occur within the mosquito, it is not surprising that climatological covariates that are most clearly associated with mosquito ecology have been linked to malaria metrics. Rainfall and temperature, measured in a variety of ways, have been found to be significant drivers of malaria considerably more than any other covariate ($34\,\%$, 54/159 and $40.3\,\%$, 64/159, respectively).

Despite the an increase in the spatial and temporal resolution of explanatory covariates, as noted previously, existing data are often inadequate to predict mosquito abundance at the fine spatial scale upon which mosquito population dynamics occur. For example, measured either at a local weather station or through satellite derived metrics, it is unclear how to translate a single ‘rainfall’ data location to predict the presence and quantity of larval breeding sites. Satellite-derived vegetation indices, such as NDVI, have been demonstrated to be useful to measure landscape suitability for mosquitoes ($13.8\,\%$, 4/29), but in the literature they have been shown only to correlate concurrently to abundance (or at most lagged one month). In general, remotely-sensed data provide an opportunity for increased understanding, but their utility (and accuracy) is tempered by potentially complex, confounding variables such as land-type. For example, high NDVI values can indicate very different climates depending on whether the region measured has irrigation, is heavily forested or is on the desert fringe. Likewise, due to local variation in land-type, the same amount of rainfall can have a very different impact on mosquito larval sites depending on where it is measured.

There are (at least) three different time-scales of malaria metrics, those related to mosquito population dynamics, those related to malaria incidence and those related to malaria prevalence. As the time-scale of the metric increases, and the lag between occurrence of a driver of transmission and the time its effect is felt upon the given metric increases, the complexity of the relationship likewise increases. The response of mosquito population dynamics to climatological forcing is essentially instantaneous. In addition to a non-linear relationship to temperature, the necessity of rainfall for larval sites combined with the hazard of flushing of these sites by flooding associated with heavy rainfall introduces a second non-linear relationship between climate and ‘malaria’ vis-à-vis mosquito density. Translating the climatic effects through mosquito density, two blood meals (one infecting the mosquito and a second infecting a susceptible host) and the ‘incubation’ period in a human between initial infecting bite and the development of clinical symptoms clearly temporally separates human incidence and climate drivers. Adding to this complexity, climatic drivers such as temperature have been shown to influence incubation periods. Thus, the second scale of drivers is based on malaria data associated with incidence (e.g. case data, death). The longest scales of metrics are associated with prevalence. Integrating the amount of incidence across an entire transmission season, and then incorporating the waning of immunity that will slowly decrease the contribution of early infections to later prevalence surveys, these malaria variables are the least immediately influenced by season. Beyond the expectation of three different temporal scales of climate influence on malaria, different challenges are involved with measuring each of these malaria metrics. Those most likely to be greatly influenced by climate (e.g., mosquito abundance) are also the most stochastic and require the most serial samples to accurately account for measurement noise. Likewise, although incidence is the most practically important measure of malaria transmission, it is unclear if analyses that only focus on the relationship between climatological covariates and incidence would be most useful to predict the seasonality of incidence. Rather, analyses of the drivers of seasonal fluctuations of measures of the potential of transmission (e.g., mosquito abundance) may be more indicative of the underlying seasonality of malaria.

Perhaps due to the relative simplicity of the corresponding data analysis, or perhaps due to the noise reduction that occurs when taking means, synoptic data (i.e., using the mean of a metric for a given month across study years as the expected value for that month) have been used extensively to assess both seasonal patterns of malaria as well as the effects of climatological covariates on malaria data. In a sense, the synoptic curve of incidence in a location is a close proxy to the seasonal pattern of malaria transmission intensity within the region. Were there to exist no inter-annual variation in incidence (or drivers) these two quantities would be comparable. As such, to infer a basic level of understanding of seasonal patterns, synoptic data can be a useful tool. However, in reality the previous premise is demonstrably false. Natural, intrinsic periodicity in malaria transmission suggests that averaging over years to produce a single value for expected incidence on a given day (or, more commonly, in a given month) obfuscates the true annual patterns and may bias inference [45]. Further, if climate is closely linked to incidence, averaging incidence across years with vastly different rainfall or temperature may result in producing seasonal signatures that, in practice, never occur themselves. Finally, global drivers of climate like ENSO have multi-year cycles and synoptic data implicitly ignore any potential impact of these sorts of covariates. Removing all influence of climatological drivers, or even more extreme all extrinsic drivers, would likely remove much of the crucial seasonal patterns that are needed to inform public policy. Instead, accounting for the *inter-annual* variation that can be ascribed to external drivers would still leave the necessary seasonal patterns. Such an analysis may result in a signal much different from that of the synoptic mean, and as such de-trending the data must be done with considerable care.

The analysis conducted by the papers reviewed was typically strongly driven by the question the study was designed to address. Because most of the studies included in this review were not focused on assessing the strength and signal of seasonality, the types of analyses were not directed to this task. The most frequent purpose of a study was to link climatological covariates to temporal variation in a specific malaria metric. This variation was acknowledged to occur at both intra- and inter-annual scales, but beyond fine-scale temporal variation, the studies most frequently focused on inter-annual scales. For the mechanistic approaches, except for a few that investigated the intrinsic periodic properties of their system, seasonality was incorporated by including relationships between parameters and climatological and temporal covariates. The most frequent driver of temporal variation in these studies concerned the daily survival rate of the mosquito. A non-linear relationship [18] has been identified in laboratory and field studies, where mosquitoes are more likely to die at both extremely cold and extremely hot temperatures. Recent work by Mordecai et al. have shown that non-linear temperature responses in all mosquito and parasite parameters that determine malaria transmission.

The scope of this review concerns the current seasonal patterns of malaria across the globe. Although it is, thus, outside the purview of this review, the growing literature assessing the potential changes in the range and incidence of malaria in the face of potential changes in local and global climate must be noted. Within our review, 51 publications were excluded from further analysis because they were identified as being solely concerned with assessing some aspect of the impact of climate change on malaria. Many of these works have combined the predicted climate maps produced by WorldClim or ClimMond [46] with the mosquito daily survival rates identified by Martens and others to predict either changes in range of climate suitability (which does not always imply ‘increases’ in range) or changes in incidence vis-à-vis changes in the length of the year for which transmission is possible. Given the extremely complex interplay between the natural transmission dynamics of malaria and the impact that humans and economic development exert on the system (either positively or negatively), understanding the consequences of a 5 °C increase in local temperature on malaria remains a pertinent, but poorly understood problem.

It is important to note that several previous studies have paved the way for this comprehensive review and have, themselves, begun the effort in earnest to quantify seasonal patterns either locally or in large regions across the globe. Mabaso et al. [47] applied Markham’s concentration index [48] to data from Zimbabwe. They identified significant effects of both temperature and rainfall in determining the strength and timing of seasonal outbreaks. Roca et al. [5] conducted a systematic literature review of studies concerning the age of paediatric hospital admissions with severe malaria syndromes. This was followed by estimation of the potential impact of seasonal malaria chemoprevention on children across Africa [2], work which suggested that seasonal prevention strategies could avert millions of malaria cases and tens of thousands of childhood deaths every year. Ermert et al. [40] utilized the Liverpool model [30] to approximate seasonality by identifying when the estimated EIR in a location first exceeded 0.1. They also were able to reliably recreate seasonal quantities such as the beginning and end of the ‘season’ with their model when applied to West Africa. Gemperli et al. [21] used a seasonality map derived from climatological covariates (rainfall, temperature and NDVI) within a mechanistic modelling framework to estimate the length of the malaria season. Each of these studies, as well as several others, has indicated that there appears to be some level of predictability of malaria seasonality in endemic settings.

While these and other studies have investigated aspects of seasonality, either synoptically over large regions or in depth locally, the drivers and patterns of seasonality at the global level remain poorly understood. Malaria seasonality, though difficult itself to fully describe quantitatively, is not measurable from a single years’ transmission pattern. The confounding and driving nature of climatological covariates requires a multi-faceted modelling approach. Both statistically and mechanistically, parsing the relative contribution of climate and an underlying seasonal pattern to observed data requires acquiring data with a minimal amount of measurement error or in sufficient quantities to reduce prediction error. Further, linking the patterns observed or identified in one specific location to the surrounding area and understanding the uncertainty in the extrapolated patterns of seasonality in the locations where data are scarce is critical. Both statistical and mechanistic approaches provide useful (and different) information and, thus, both should be used in concert to most adequately exploit the available data. Further, although there have been numerous studies that created extremely useful data, this data must be made openly available to allow for the type of synthetic analysis required to inform public policy. It would appear that only by modelling seasonal patterns at both small and large spatial scales while incorporating the inter-annual variability introduced by capricious climatological drivers can a clear picture of malaria seasonality be understood.

## Additional files

Additional file 1: Number of studies by location and metric Additional file 2: Number of studies by climate driver and metric (minimum and maximum significant lag identified in brackets). Additional file 3: Number of studies by modeling approach and metric Additional file 4: Number of statistical studies by metric. Additional file 5: Number of studies by modeling approach and driver. Additional file 6: Number of statistical studies by driver. Additional file 7: Number of studies by location and climate driver. Additional file 8: Number of studies by location and modeling approach. Additional file 9: Number of statistical studies by location. Additional file 10: Mean lag identified (standard error in parentheses) by location and climate driver for mosquito abundance. Additional file 11: Mean lag identified (standard error in parentheses) by location and climate driver for incidence. Additional file 12: Mean lag identified (standard error in parentheses) by location and climate driver for EIR. Additional file 13: Mean lag identified (standard error in parentheses) by location and climate driver for prevalence. Additional file 14: Systematic review raw data. Additional file 15: Systematic review additional detailed analysis. Additional file 16: Number of publications concerning seasonality of malaria by year Temporal trend in the publication of models included in the bibliography, grouped by modeling approach and binned by five-year period. “Both” represents papers that consider both a mechanistic and statistical modeling approach within the same publication. Additional file 17: Global distribution of malaria seasonality papers using mechanistic models. The frequency of the use of mechanistic models for studying malaria seasonality by country is plotted. Additional file 18: Distribution of malaria seasonality papers by malaria metric. The frequency with which different proxies of malaria transmission were studied is plotted for (panel A) mosquito abundance, (panel B) incidence, (panel C) EIR and (panel D) prevalence. Additional file 19: Systematic review complete list of manuscripts reviewed.
