# Supplementary material for: Seasonality of Plasmodium falciparum transmission: a systematic review
Source: Malar J. 2015 Sep 15;14:343. doi: 10.1186/s12936-015-0849-2 (PMC4570512; doi:10.1186/s12936-015-0849-2)
Supplement: Additional file 1: — Number of studies by location and metric [file 12936_2015_849_MOESM1_ESM.pdf]

Number of studies by location and metric.

	Mosquito Abundance	Incidence	EIR	Prevalence	Other	Total
Regions of Africa						
Africa	0	1	0	0	4	5
African Highlands	1	2	0	0	0	2
Central Africa	0	0	0	1	0	1
Sub-saharan Africa	0	0	1	0	1	2
West Africa	0	2	0	2	0	4
Specific Countries in Africa						
Benin	1	0	1	1	1	4
Botswana	0	3	0	0	0	3
Burkina Faso	1	1	0	2	2	6
Burundi	0	3	0	0	0	3
Cameroon	0	1	0	0	0	1
Eritrea	0	2	0	1	0	3
Ethiopia	0	6	0	0	1	7
Gambia	1	0	0	1	0	2
Ghana	2	1	0	1	2	5
Guinea-Bissau	1	0	0	0	0	1
Ivory Coast	0	0	0	1	1	2
Kenya	4	6	3	1	5	17
Liberia	0	0	0	1	0	1
Madagascar	0	0	0	1	0	1
Malawi	0	1	0	0	2	3
Mali	2	1	0	2	0	5
Mozambique	0	3	0	0	1	4
Niger	3	0	0	0	1	4
Nigeria	1	1	0	0	1	3
Senegal	1	0	0	2	1	4
Sierra Leone	1	0	0	1	0	2
South Africa	0	4	0	0	0	4
Sudan	1	3	0	1	0	5
Tanzania	3	3	1	2	2	10
Togo	1	0	0	1	0	2
Zimbabwe	0	2	0	0	0	2
Regions of Asia						
East Asia	0	0	0	0	1	1
South Asia	0	0	0	0	1	1
Specific Countries in Asia						
Bangladesh	2	2	0	0	0	4
China	0	7	0	0	1	8
India	0	4	0	0	0	4
Iran	0	1	0	0	1	2
South Korea	0	1	0	0	1	2
Sri Lanka	0	2	0	0	0	2
Thailand	1	1	0	0	1	3
Vietnam	0	1	0	0	0	1
Specific Countries of Europe						
Poland	0	0	0	0	1	1
Portugal	1	0	0	0	0	1
Specific Countries in South America						
Brazil	1	2	0	0	2	5
Colombia	0	2	0	0	0	3
Honduras	0	1	0	0	0	1
Nicaragua	0	1	0	0	0	1
Paraguay	0	1	0	0	0	1
Total	29	72	6	22	34	159
