# Supplementary material for: Seasonality of Plasmodium falciparum transmission: a systematic review
Source: Malar J. 2015 Sep 15;14:343. doi: 10.1186/s12936-015-0849-2 (PMC4570512; doi:10.1186/s12936-015-0849-2)
Supplement: Additional file 2: — Number of studies by climate driver and metric (minimum and maximum significant lag identified in brackets). [file 12936_2015_849_MOESM2_ESM.pdf]

Number of studies by climate driver and metric (minimum and maximum significant lag identified in brackets).

	Mosquito Abundance	Incidence	EIR	Prevalence	Other	Total
Rainfall	4 [1-2]	37 [0-6]	2 [1-2]	4 [0-0]	8	54
Temperature	20 [0-1]	36 [0-9]	4 [0-1]	4 [0-2]	5	64
Vegetation Indicies	4 [0-0]	9 [0-3]	2 [0-0]	3 [0-1]	1	18
Other	3	18	2	2	1	24
Total	29	72	6	22	34	159
