# Supplementary material for: Seasonality of Plasmodium falciparum transmission: a systematic review
Source: Malar J. 2015 Sep 15;14:343. doi: 10.1186/s12936-015-0849-2 (PMC4570512; doi:10.1186/s12936-015-0849-2)
Supplement: Additional file 3: — Number of studies by modeling approach and metric [file 12936_2015_849_MOESM3_ESM.pdf]

Number of studies by modeling approach and metric.

	Mosquito Abundance	Incidence	EIR	Prevalence	Other	Total
Statistical	23	58	5	22	20	125
Mechanistic	6	18	1	2	5	31
Other	0	0	0	0	8	8
Total	29	72	6	22	34	159
