# Supplementary material for: Seasonality of Plasmodium falciparum transmission: a systematic review
Source: Malar J. 2015 Sep 15;14:343. doi: 10.1186/s12936-015-0849-2 (PMC4570512; doi:10.1186/s12936-015-0849-2)
Supplement: Additional file 4: — Number of statistical studies by metric. [file 12936_2015_849_MOESM4_ESM.pdf]

Number of statistical studies by metric.

	Mosquito Abundance	Incidence	EIR	Prevalence	Other	Total
Simple	4	12	1	2	6	22
Regression	14	23	1	3	9	50
Spatial and/or Bayesian	4	9	1	14	4	31
Total	29	72	6	22	34	159
