# Supplementary material for: Seasonality of Plasmodium falciparum transmission: a systematic review
Source: Malar J. 2015 Sep 15;14:343. doi: 10.1186/s12936-015-0849-2 (PMC4570512; doi:10.1186/s12936-015-0849-2)
Supplement: Additional file 5: — Number of studies by modeling approach and driver. [file 12936_2015_849_MOESM5_ESM.pdf]

Number of studies by modeling approach and driver.

	Rainfall	Temperature	Vegetation Indices	Other	Total
Statistical	51	56	18	24	125
Mechanistic	7	12	2	0	31
Other	0	0	0	0	8
Total	54	64	18	24	159
