# Supplementary material for: Seasonality of Plasmodium falciparum transmission: a systematic review
Source: Malar J. 2015 Sep 15;14:343. doi: 10.1186/s12936-015-0849-2 (PMC4570512; doi:10.1186/s12936-015-0849-2)
Supplement: Additional file 6: — Number of statistical studies by driver. [file 12936_2015_849_MOESM6_ESM.pdf]

Number of statistical studies by driver.

	Rainfall	Temperature	Vegetation Indices	Other	Total
Simple	8	6	3	1	22
Regression	18	25	7	8	50
Spatial and/or Bayesian	15	15	6	7	31
Total	41	46	16	16	103
