# Supplementary material for: Seasonality of Plasmodium falciparum transmission: a systematic review
Source: Malar J. 2015 Sep 15;14:343. doi: 10.1186/s12936-015-0849-2 (PMC4570512; doi:10.1186/s12936-015-0849-2)
Supplement: Additional file 7: — Number of studies by location and climate driver. [file 12936_2015_849_MOESM7_ESM.pdf]

Number of studies by location and climate driver.

	Rainfall	Temperature	Vegetation Indices	Other	Total
Regions of Africa					
Africa	3	3	0	1	5
African Highlands	1	2	0	0	2
Central Africa	1	1	1	0	1
Sub-saharan Africa	0	1	0	1	2
West Africa	2	2	2	1	4
Specific Countries in Africa					
Benin	1	2	1	0	4
Botswana	2	1	0	0	3
Burkina Faso	1	2	0	0	6
Burundi	2	2	0	0	3
Cameroon	1	1	0	0	1
Eritrea	2	1	2	1	3
Ethiopia	5	5	1	1	7
Gambia	0	1	0	0	2
Ghana	0	2	0	0	5
Guinea-Bissau	0	1	0	0	1
Ivory Coast	0	0	0	0	2
Kenya	5	5	2	3	17
Liberia	0	0	0	0	1
Madagascar	0	0	0	0	1
Malawi	3	0	0	0	3
Mali	1	2	2	0	5
Mozambique	0	2	0	3	4
Niger	0	1	0	0	4
Nigeria	0	1	0	1	3
Senegal	0	1	0	0	4
Sierra Leone	0	1	0	0	2
South Africa	1	1	0	0	4
Sudan	2	1	1	1	5
Tanzania	4	2	0	0	10
Togo	0	1	0	0	2
Zimbabwe	2	2	1	1	2
Regions of Asia					
East Asia	0	1	0	0	1
South Asia	0	0	0	0	1
Specific Countries in Asia					
Bangladesh	1	1	3	0	4
China	4	7	0	4	8
India	2	0	1	0	4
Iran	1	2	0	2	2
South Korea	1	1	0	1	2
Sri Lanka	2	0	0	0	2
Thailand	2	2	0	0	3
Vietnam	1	1	0	1	1
Specific Countries of Europe					
Poland	0	0	0	0	1
Portugal	0	1	0	0	1
Specific Countries in South America					
Brazil	1	0	0	0	5
Colombia	0	1	0	2	3
Honduras	0	0	0	0	1
Nicaragua	0	0	0	0	1
Paraguay	0	0	1	0	1
Total	54	64	18	24	159
