# Supplementary material for: Seasonality of Plasmodium falciparum transmission: a systematic review
Source: Malar J. 2015 Sep 15;14:343. doi: 10.1186/s12936-015-0849-2 (PMC4570512; doi:10.1186/s12936-015-0849-2)
Supplement: Additional file 8: — Number of studies by location and modeling approach. [file 12936_2015_849_MOESM8_ESM.pdf]

Number of studies by location and modeling approach.

	Statistical	Mechanistic	Other	Total
Regions of Africa				
Africa	4	0	1	5
African Highlands	1	1	0	2
Central Africa	1	1	0	1
Sub-saharan Africa	1	0	1	2
West Africa	2	3	0	4
Specific Countries in Africa				
Benin	4	0	0	4
Botswana	3	1	0	3
Burkina Faso	4	1	0	6
Burundi	3	0	0	3
Cameroon	1	1	0	1
Eritrea	3	0	0	3
Ethiopia	7	0	0	7
Gambia	2	0	0	2
Ghana	4	0	1	5
Guinea-Bissau	1	0	0	1
Ivory Coast	1	0	1	2
Kenya	14	3	0	17
Liberia	1	0	0	1
Madagascar	1	0	0	1
Malawi	3	0	0	3
Mali	5	0	0	5
Mozambique	4	0	0	4
Niger	1	3	0	4
Nigeria	2	1	0	3
Senegal	3	0	1	4
Sierra Leone	2	0	0	2
South Africa	2	2	0	4
Sudan	5	0	0	5
Tanzania	7	3	0	10
Togo	2	0	0	2
Zimbabwe	1	1	0	2
Regions of Asia				
East Asia	0	1	0	1
South Asia	0	1	0	1
Specific Countries in Asia				
Bangladesh	4	0	0	4
China	8	0	0	8
India	4	2	0	4
Iran	2	0	0	2
South Korea	1	0	1	2
Sri Lanka	2	0	0	2
Thailand	2	0	1	3
Vietnam	1	0	0	1
Specific Countries of Europe				
Poland	1	0	0	1
Portugal	0	1	0	1
Specific Countries in South America				
Brazil	2	2	1	5
Colombia	2	1	0	3
Honduras	0	1	0	1
Nicaragua	0	1	0	1
Paraguay	1	0	0	1
Total	125	31	8	159
