# Supplementary material for: Seasonality of Plasmodium falciparum transmission: a systematic review
Source: Malar J. 2015 Sep 15;14:343. doi: 10.1186/s12936-015-0849-2 (PMC4570512; doi:10.1186/s12936-015-0849-2)
Supplement: Additional file 9: — Number of statistical studies by location. [file 12936_2015_849_MOESM9_ESM.pdf]

Number of statistical studies by location.

	Simple	Regression	Spatial and/or Bayesian	Total
Regions of Africa				
Africa	0	1	1	2
African Highlands	0	1	0	1
Central Africa	0	0	1	1
Sub-saharan Africa	0	1	0	1
West Africa	0	0	2	2
Specific Countries in Africa				
Benin	0	2	1	3
Botswana	0	0	0	0
Burkina Faso	2	3	1	6
Burundi	0	1	2	3
Cameroon	0	0	0	0
Eritrea	1	1	0	2
Ethiopia	1	6	0	7
Gambia	0	1	1	2
Ghana	2	1	1	4
Guinea-Bissau	0	1	0	1
Ivory Coast	0	0	1	1
Kenya	3	3	1	7
Liberia	0	0	1	1
Madagascar	0	0	0	0
Malawi	0	2	1	3
Mali	1	2	2	5
Mozambique	1	0	3	4
Niger	0	1	0	1
Nigeria	0	1	0	1
Senegal	1	1	1	3
Sierra Leone	0	1	1	2
South Africa	0	2	0	2
Sudan	1	1	1	3
Tanzania	2	4	1	7
Togo	0	1	1	2
Zimbabwe	0	0	1	1
Regions of Asia				
East Asia	0	0	0	0
South Asia	0	0	0	0
Specific Countries in Asia				
Bangladesh	0	3	1	4
China	1	5	1	7
India	3	0	0	3
Iran	0	1	1	2
South Korea	0	1	0	1
Sri Lanka	1	0	1	2
Thailand	1	0	0	1
Vietnam	0	0	1	1
Specific Countries of Europe				
Poland	0	1	0	1
Portugal	0	0	0	0
Specific Countries in South America				
Brazil	1	0	1	2
Colombia	0	1	0	1
Honduras	0	0	0	0
Nicaragua	0	0	0	0
Paraguay	0	0	0	0
Total	22	50	31	103
