# Supplementary material for: Seasonality of Plasmodium falciparum transmission: a systematic review
Source: Malar J. 2015 Sep 15;14:343. doi: 10.1186/s12936-015-0849-2 (PMC4570512; doi:10.1186/s12936-015-0849-2)
Supplement: Additional file 10: — Mean lag identified (standard error in parentheses) by location and climate driver for mosquito abundance. [file 12936_2015_849_MOESM10_ESM.pdf]

Mean lag identified (standard error in parentheses) by location and climate driver for mosquito abundance.

	Rainfall	Temperature	Vegetation Indices
Regions of Africa			
African Highlands	-	Mech.	-
Specific Countries in Africa			
Benin	-	0 (NA)	-
Burkina Faso	-	0 (NA)	-
Gambia	-	0 (NA)	-
Ghana	-	0 (0)	-
Guinea-Bissau	-	0 (NA)	-
Kenya	2 (NA)	0.33 (0.58)	0 (NA)
Mali	1.5 (0.71)	0 (0)	0 (NA)
Niger	-	0 (NA)	-
Nigeria	-	0 (NA)	-
Senegal	-	0 (NA)	-
Sierra Leone	-	0 (NA)	-
Tanzania	0 (NA)	0 (0)	-
Togo	-	0 (NA)	-
Specific Countries in Asia			
Bangladesh	-	-	0 (0)
Thailand	0 (NA)	0 (NA)	-
Specific Countries of Europe			
Portugal	-	0 (NA)	-
