# Supplementary material for: Seasonality of Plasmodium falciparum transmission: a systematic review
Source: Malar J. 2015 Sep 15;14:343. doi: 10.1186/s12936-015-0849-2 (PMC4570512; doi:10.1186/s12936-015-0849-2)
Supplement: Additional file 11: — Mean lag identified (standard error in parentheses) by location and climate driver for incidence. [file 12936_2015_849_MOESM11_ESM.pdf]

Mean lag identified (standard error in parentheses) by location and climate driver for incidence.

	Rainfall	Temperature	Vegetation Indices
Regions of Africa			
Africa	3 (NA)	0 (NA)	-
African Highlands	1.5 (0.71)	3.5 (1.29)	-
Specific Countries in Africa			
Botswana	2.25 (1.71)	3 (1)	-
Burkina Faso	0 (NA)	Mech.	-
Burundi	0 (0)	0 (0)	-
Cameroon	3 (1)	NA (NA)	-
Eritrea	2.5 (0.58)	-	0.33 (0.58)
Ethiopia	2.23 (0.86)	1.96 (1.32)	3 (NA)
Ghana	-	0 (NA)	-
Kenya	2.89 (1.9)	0 (0)	-
Malawi	1 (NA)	-	-
Mali	-	-	0.5 (NA)
Mozambique	-	0 (0)	-
South Africa	Mech.	5 (2.74)	-
Sudan	0.5 (0.71)	0 (NA)	2.5 (NA)
Tanzania	2 (NA)	0 (NA)	-
Zimbabwe	3 (1.41)	3 (1.41)	2 (NA)
Specific Countries in Asia			
Bangladesh	0 (NA)	0 (NA)	1.5 (1.29)
China	1.67 (0.82)	1.56 (1.13)	-
India	1 (0)	-	2 (1)
Iran	-	1 (NA)	-
South Korea	2.5 (NA)	1.25 (0.71)	-
Sri Lanka	1.5 (1.29)	-	-
Thailand	0.5 (0.71)	0 (NA)	-
Vietnam	0 (NA)	0 (NA)	-
Specific Countries in South America			
Brazil	0 (NA)	-	-
Colombia	-	Mech.	-
Paraguay	-	-	0 (NA)
