# Supplementary material for: Seasonality of Plasmodium falciparum transmission: a systematic review
Source: Malar J. 2015 Sep 15;14:343. doi: 10.1186/s12936-015-0849-2 (PMC4570512; doi:10.1186/s12936-015-0849-2)
Supplement: Additional file 12: — Mean lag identified (standard error in parentheses) by location and climate driver for EIR. [file 12936_2015_849_MOESM12_ESM.pdf]

Mean lag identified (standard error in parentheses) by location and climate driver for EIR.

	Rainfall	Temperature	Vegetation Indices
Regions of Africa			
Sub-saharan Africa	-	0 (NA)	-
Specific Countries in Africa			
Benin	-	Mech.	-
Kenya	1.5 (0.71)	0.33 (0.58)	0 (0)
