# Supplementary material for: Seasonality of Plasmodium falciparum transmission: a systematic review
Source: Malar J. 2015 Sep 15;14:343. doi: 10.1186/s12936-015-0849-2 (PMC4570512; doi:10.1186/s12936-015-0849-2)
Supplement: Additional file 13: — Mean lag identified (standard error in parentheses) by location and climate driver for prevalence. [file 12936_2015_849_MOESM13_ESM.pdf]

Mean lag identified (standard error in parentheses) by location and climate driver for prevalence.

	Rainfall	Temperature	Vegetation Indices
Regions of Africa			
Central Africa	0 (NA)	1.5 (0.71)	1 (NA)
West Africa	0 (0)	1 (0.82)	0.5 (0.71)
Specific Countries in Africa			
Eritrea	-	0 (NA)	-
Tanzania	2 (1)	-	-
