# Supplementary material for: Seasonality of Plasmodium falciparum transmission: a systematic review
Source: Malar J. 2015 Sep 15;14:343. doi: 10.1186/s12936-015-0849-2 (PMC4570512; doi:10.1186/s12936-015-0849-2)
Supplement: Additional file 15: — Systematic review additional detailed analysis. [file 12936_2015_849_MOESM15_ESM.pdf]

# Seasonality of *Plasmodium falciparum* transmission: a systematic review

Robert C. Reiner, Jr.*[1,2,3], Matthew Geary*[4], Peter M. Atkinson[5], David L. Smith[1,6,7], and Peter W. Gething[1,7]

[1]Fogarty International Center, National Institutes of Health, Bethesda, MD, USA
[2]Department of Epidemiology and Biostatistics, Indiana University School of Public Health, Bloomington, IN, USA
[3]Department of Entomology, University of California, Davis, CA, USA
[4]Department of Biological Sciences, University of Chester, Chester, UK
[5]Global Environmental Change and Earth Observation Research Group, Geography and Environment, University of Southampton, UK
[6]Center for Disease Dynamics, Economics & Policy, Washington, DC, USA
[7]Spatial Ecology and Epidemiology Group, Department of Zoology, Oxford University, Oxford, UK

## Appendix 1 Temperature

In 24 different analyses, spanning multiple decades and much of Africa and Asia, the relationship between minimum temperature and the malaria metric of interest varied depending on both space and the type of variable used as a proxy for malaria. Investigations into mosquito abundance in both western Africa and Thailand [1, 2] and mosquito size in Thailand [3] found a direct relationship between a given month's minimum temperature and mosquito abundance or development. Due to the temporal scale at which mosquito development occurs (1-2 weeks), it is not surprising that there is an immediate effect of minimum temperature on variables associated with mosquito development. Contrastingly, a range of lags was found to be significant between minimum monthly temperature and direct measures of malaria (e.g. case data, malaria morbidity, etc.). The lag found to be most significant between minimum temperature ranged from 0

*These authors contributed equally to this work.

months in countries such as Iran [4] and Burundi [5, 6] up to 5 months in Eastern Africa, specifically the East African Highlands [7] and Ethiopia [8]. In all but one instance, the relationship between minimum monthly temperature was found to be positive; in Mali, there was a significant negative relationship between the abundance of two mosquito vectors and the coinciding month's minimum temperature. As could be expected, many of the investigations that found a significant effect of minimum temperature also tested maximum temperature (a relationship that was not as true in the converse). Several studies discarded maximum temperature as a significant driver when tested in a model that already contained minimum temperature [e.g. [9, 10]], but others noted the co-linearity between the two variables (minimum and maximum monthly temperature) and just selected one of them for further testing [11].

Presumably, at least partially due to several empirical results about the effect of high temperatures on mosquito lifespan and suitability in lab settings, maximum temperature was the most frequently tested temperature covariate. In 19 different studies, maximum temperature was found to be a significant driver of malaria or a proxy for malaria. As with minimum temperature, the lag identified as 'optimal' depended heavily on the response variable within the study. Studies that correlated temperature to malaria cases (14) varied greatly in the reported lag between maximum temperature and cases, ranging from 0 months in China [12] and Ethiopia [8] up to 5 months in the African highlands [7]. Of these studies, six simultaneously found minimum temperature as a significant driver; only one study in southwest China found a different significant lag for minimum temperature (1 and 2 months) and maximum temperature (4 months) [13]. Also in agreement with studies that found minimum temperature to be a significant driver of mosquito abundance, there was an instantaneous effect of maximum temperature found mosquito development rates in Tanzania, Ghana and Kenya [14]. Only one study each identified a significant lag between maximum temperature and malaria prevalence [15] or malaria morbidity [6]. In both cases, an instant effect of maximum temperature was identified.

Eight studies identified mean monthly temperature as an important driver. Incidence was correlated with mean temperature in five studies, and again results varied greatly. In South Africa significant lags between mean temperature and incidence were identified up to 9 months in the past [16], while in Thailand the effect was instantaneous [17]. A small number of studies investigated other temperature-related quantities, such as degree-days and temperature indices based on previous work. In each of these few cases, all related to mosquito development, there was no lag between the malarial covariate and the temperature-based driver.

## Appendix 2 Rainfall

The majority of the studies that identified rainfall as a significant driver of malaria concentrated on African and Asian locations with only one concentrating on South America [18]. The maximum lag used for rainfall variables was four months in East Africa. Lag values were generally longer in Africa and shorter in Asia (Additional File 17). Contrastingly, one study in Sri Lanka used a three month lag [19] and several African studies used predictors with no lag. The majority of malaria measures predicted by rainfall were counts of cases or measures of malaria influence. Because these measures dominated the studies, examples range from long lags to no lag at all. Studies using EIR and vector density were less heavily represented within these studies and, where used, the rainfall predictors tended to have shorter lags (e.g. [20]). The most common variable to be used alongside rainfall as a predictor was some form of temperature metric (21 papers) followed by NDVI (5 papers) or both together (3 papers). However, rainfall was used as a lone predictor in 14 analyses. Many papers noted that both temperature and rainfall might affect vector reproduction and development, but only one [7] noted that, for their study area, rainfall or temperature alone was not enough to describe malaria trends.

Of the studies using rainfall metrics to predict malaria variables, the most common precipitation measure was mean monthly rainfall, which was used by ten papers. Most of these papers also used temperature as a predictor variable along with three which used NDVI and one which used variables such as a soil water index and distance to water. Along with mean monthly rainfall [21] also found a relationship between seasonal indices of both malaria and rainfall. [22] and [23] used a broader characterisation of seasonality by using the season itself (wet or dry) as a predictor of malaria morbidity. One paper [20], focusing on mosquito density, used a short-term mean between their two study periods and two papers used a weekly mean [24, 25], with a combination of lag values for the predictor variables. Seven of these analyses used total rainfall to predict malaria metrics. Of these, three papers used temperature variables, four used NDVI, and one of these found NDVI to be a more accurate predictor than rainfall [26]. Along with these common predictors one study used influence of Indian Ocean Dipole in conjunction with total monthly rainfall and one study used total monthly rainfall alone, but found little significance due to the climate of the area [27].

Several other interesting approaches used rainfall as a predictor of malaria metrics. Abeku *et al.* [28] used the frequency of abnormal weather conditions, including monthly rainfall, to investigate the relationship with epidemic events in the Ethiopian rift valley. Ceccato *et al.* [29] also calculated anomalous rainfall events and used both satellite and weather station rainfall data. Where available, weather station rainfall data were found to be a more accurate predictor of malaria cases. Teklehaimanot *et al.* [24, 25] investigated malaria cases in Ethiopia and used weekly rainfall data along with minimum and maximum temperature in several districts. They tried lags ranging from 4-12 weeks and found that relationships between malaria cases and rainfall changed depending on the lag time and district of interest. Gosoniu *et al.* [15] also spanned several locations, in West Africa on this occasion, and found that different meteorological predictors (e.g. mean, min, max or total rainfall, NDVI and temperature) were more suitable for

predicting malaria prevalence in different areas. Other multi-location studies included [8] who also found that different predictors were included in the best performing models in different areas. Rainfall was included in models for four out of the 23 areas and was lagged for two months in one location and three in another to predict malaria cases in Ethiopia. Thomson *et al.* [30] is also a multi-location study using rainfall data in a discussion and partial demonstration of an early warning system for malaria using DEMETER. In contrast to the above studies using monthly, or longer, data and multiple locations [20] used a single location with daily visits to investigate weekly biting rate and EIR in Kisian, Kenya. Their model included maximum and minimum temperature, NDVI and soil moisture along with *in situ* precipitation measurements. Precipitation alone was a limited predictor of human biting rate but lagged for 2-4 weeks and used in conjunction with minimum temperature it was more predictive. Soil moisture was a more effective predictor of EIR.

Rather than a rainfall time-series or lagged rainfall alone, some studies used specific rainfall values in their models of malaria metrics. Craig *et al.* [16] and Pascual *et al.* [31] both used the rainfall recorded during a set time prior to the malaria season. Craig *et al.* [16] used the total rainfall during the summer period along with a lagged temperature variable to predict malaria cases in South Africa whereas Pascual *et al.* [31] used the total rainfall from the previous winter (November-January) to predict malaria cases in Kenya. Two further papers used rainfall from a particular period but in their studies, this period was variable. Beguin *et al.* [32] used the mean precipitation from the wettest month and Kulkarni *et al.* [33] used the mean rainfall in the warmest and coldest months; both papers were attempting to predict malaria distributions. Rather than rainfall itself as a predictor, Monteirode Barros *et al.* [18] used the number of wet days as a predictor of vector biting rate in their study in Brazil finding that from the number of wet days, monthly rainfall, adult densities, degree of wetness, temperature and humidity, wet days was the only significant predictor of biting rate.

## Appendix 3 Vegetation Index

The majority of the papers that found a vegetation index to be a significant driver of malaria metrics used NDVI. Of those that used NDVI, the majority of studies used monthly maximum NDVI, some used mean NDVI (e.g. Sogoba *et al.* [2]) and Gosoniu *et al.* [15] used a combination of the two. One paper used the Enhanced Vegetation Index (EVI; [34]) along with Evapotranspiration index (ETa) and two papers [35, 36] used the vegetation and temperature condition indices (VCI and TCI respectively). Eight of the sixteen papers using vegetation indices used the number of malaria cases as their response variable. Two papers used malaria prevalence as their predictor [37, 15] and one used incidence of malaria [38]. Five of the papers were more closely focused on mosquitoes carrying malaria. Two papers attempted to predict EIR. One of these also investigated weekly biting rate and the other vector density. Two further papers used vector density alone and one paper used the relative frequency of two malaria vectors in Mali [2].

Half of the papers in this group lagged the vegetation index and all but one [34] of these papers used NDVI. None of the studies which focused on malaria vectors rather than disease metrics used lagged vegetation indices. The longest lags were found in India [26] and Bangladesh [39] using NDVI and Ethiopia [34] using EVI and ETa. Of the papers investigating malaria cases, two did not lag the vegetation index. These studies were located in Eritrea and Paraguay and both used NDVI as a predictor. One paper investigating malaria prevalence in West Africa used NDVI with no lag. The majority of the papers used vegetation indices as predictors in their own right but some papers used them as proxies for other variables. Creasey *et al.* [40] used NDVI as a surrogate for rainfall and Gaudart *et al.* [38] used NDVI as a proxy for several environmental variables, especially relative humidity. Ceccato *et al.* [29] used the relationship between NDVI and other environmental variables in the opposite direction and tried to predict

NDVI values using rainfall data. Other studies used vegetation indices in conjunction with other environmental predictors, the most common being rainfall, as discussed above. In general, an increase in NDVI was associated with an increase in malaria metric. However, Haque *et al.* [39] found the opposite relationship in Bangladesh. This was explained because in this region mosquitoes require dry periods so that rivers dry up to form pools in which they can breed.

## References


[1] Bayoh, M.N., Thomas, C.J., Lindsay, S.W.: Mapping distributions of chromosomal forms of *Anopheles gambiae* in West Africa using climate data. Medical and Veterinary Entomology **15**(3), 267–274 (2001)

[2] Sogoba, N., Vounatsou, P., Bagayoko, M.M., Doumbia, S., Dolo, G., Gosoniu, L., Traore, S.F., Toure, Y.T., Smith, T.: The spatial distribution of *Anopheles gambiae sensu stricto* and *An. arabiensis* (Diptera : Culicidae) in Mali. Geospatial Health **1**(2), 213–222 (2007)

[3] Kitthawee, S., Edman, J.D., Upatham, E.S.: Relationship between female anopheles-dirus (diptera, culicidae) body size and parity in a biting population. Journal of Medical Entomology **29**(6), 921–926 (1992)

[4] Zayeri, F., Salehi, M., Pirhosseini, H.: Geographical mapping and Bayesian spatial modeling incidence in Sistan and Baluchistan province, Iran. Asian Pacific Journal of Tropical Medicine **4**(12), 985–992 (2011)

[5] Nkurunziza, H., Gebhardt, A., Pilz, J.: Bayesian modelling of the effect of climate on malaria in Burundi **9** (2010)

[6] Nkurunziza, H., Gebhardt, A., Pilz, J.: Geo-additive modelling of malaria in Burundi. Malaria Journal **10** (2011)

[7] Zhou, G., Minakawa, N., Githeko, A.K., Yan, G.Y.: Association between climate variability and malaria epidemics in the East African highlands. Proceedings of the National Academy of Sciences of the United States of America **101**(8), 2375–2380 (2004)

[8] Loha, E., Lindtjorn, B.: Model variations in predicting incidence of Plasmodium falciparum malaria using 1998-2007 morbidity and meteorological data from south Ethiopia. Malaria Journal **9** (2010)

[9] Abeku, T.A., De Vlas, S.J., Borsboom, G., Tadege, A., Gebreyesus, Y., Gebreyohannes, H., Alamirew, D., Seifu, A., Nagelkerke, N.J.D., Habbema, J.D.F.: Effects of meteorological factors on epidemic malaria in Ethiopia: a statistical modelling approach based on theoretical reasoning. Parasitology **128**, 585–593 (2004)

[10] Bi, P., Tong, S.L., Donald, K., Parton, K.A., Ni, J.F.: Climatic variables and transmission of malaria: A 12-year data analysis in Shuchen County, China. Public Health Reports **118**(1), 65–71 (2003)

[11] Reid, H.L., Haque, U., Roy, S., Islam, N., Clements, A.C.A.: Characterizing the spatial and temporal variation of malaria incidence in Bangladesh, 2007. Malaria Journal **11** (2012)

[12] Clements, A.C.A., Barnett, A.G., Cheng, Z.W., Snow, R.W., Zhou, H.N.: Space-time variation of malaria incidence in Yunnan province, China. Malaria Journal **8** (2009)

[13] Tian, L., Bi, Y., Ho, S.C., Liu, W., Liang, S., Goggins, W.B., Chan, E.Y.Y., Zhou, S., Sung, J.J.Y.: One-year delayed effect of fog on malaria transmission: a time-series analysis in the rain forest area of Mengla County, south-west China. Malaria Journal **7** (2008)

[14] Ikemoto, T.: Tropical Malaria Does Not Mean Hot Environments. Journal of Medical Entomology **45**(6), 963–969 (2008)

[15] Gosoniu, L., Vounatsou, P., Sogoba, N., Maire, N., Smith, T.: Mapping malaria risk in West Africa using a Bayesian nonparametric non-stationary model. Computational Statistics & Data Analysis **53**(9), 3358–3371 (2009)

[16] Craig, M.H., Kleinschmidt, I., Nawn, J.B., Le Sueur, D., Sharp, B.L.: Exploring 30 years of malaria case data in KwaZulu-Natal, South Africa: Part I. The impact of climatic factors. Tropical Medicine & International Health **9**(12), 1247–1257 (2004)

[17] Kiang, R., Adimi, F., Solka, V., Nigro, J., Singhasivanon, P., Sirichaisinthop, J., Leemingsawat, S., Apiwathnasorn, C., Looareesuwan, S.: Meteorological, environmental remote sensing and neural network analysis of the epidemiology of malaria transmission in Thailand. Geospatial Health **1**(1), 71–84 (2006)

[18] Monteiro de Barros, F.S., Honorio, N.A., Arruda, M.E.: Temporal and spatial distribution of malaria within an agricultural settlement of the Brazilian Amazon. Journal of Vector Ecology **36**(1), 159–169 (2011)

[19] Briet, O.J.T., Vounatsou, P., Amerasinghe, P.H.: Malaria seasonality and rainfall seasonality in Sri Lanka are correlated in space. Geospatial Health **2**(2), 183–190 (2008)

[20] Patz, J.A., Strzepek, K., Lele, S., Hedden, M., Greene, S., Noden, B., Hay, S.I., Kalkstein, L., Beier, J.C.: Predicting key malaria transmission factors, biting and entomological inoculation rates, using modelled soil moisture in Kenya. Tropical Medicine & International Health **3**(10), 818–827 (1998)

[21] Mabaso, M.L.H., Craig, M., Vounatsou, P., Smith, T.: Towards empirical description of malaria seasonality in southern Africa: the example of Zimbabwe. Tropical Medicine & International Health **10**(9), 909–918 (2005)

[22] Kazembe, L.N., Kleinschmidt, I., Sharp, B.L.: Patterns of malaria-related hospital admissions and mortality among Malawian children: an example of spatial modelling of hospital register data. Malaria Journal **5** (2006)

[23] Kazembe, L.N., Chirwa, T.F., Simbeye, J.S., Namangale, J.J.: Applications of Bayesian approach in modelling risk of malaria-related hospital mortality. Bmc Medical Research Methodology **8** (2008)

[24] Teklehaimanot, H.D., Lipsitch, M., Teklehaimanot, A., Schwartz, J.: Weather-based prediction of Plasmodium falciparum malaria in epidemic-prone regions of Ethiopia I. Patterns of lagged weather effects reflect biological mechanisms. Malar J **3**, 41 (2004)

[25] Teklehaimanot, H.D., Schwartz, J., Teklehaimanot, A., Lipsitch, M.: Weather-based prediction of Plasmodium falciparum malaria in epidemic-prone regions of Ethiopia II. Weather-based prediction systems perform comparably to early detection systems in identifying times for interventions. Malar J **3**, 44 (2004)

[26] Baeza, A., Bouma, M.J., Dobson, A.P., Dhiman, R., Srivastava, H.C., Pascual, M.: Climate forcing and desert malaria: the effect of irrigation. Malaria Journal **10** (2011)

[27] Kalinga-Chirwa, R., Ngongondo, C., Kalanda-Joshua, M., Kazembe, L., Pemba, D., Kululanga, E.: Linking rainfall and irrigation to clinically reported malaria cases in some villages in Chikhwawa District, Malawi. Physics and Chemistry of the Earth **36**(14-15), 887–894 (2011)

[28] Abeku, T.A., van Oortmarssen, G.J., Borsboom, G., de Vlas, S.J., Habbema, J.D.F.: Spatial and temporal variations of malaria epidemic risk in Ethiopia: factors involved and implications. Acta Tropica **87**(3), 331–340 (2003)

[29] Ceccato, P., Ghebremeskel, T., Jaiteh, M., Graves, P.M., Levy, M., Ghebreselassie, S., Ogbamariam, A., Barnston, A.G., Bell, M., del Corral, J., Connor, S.J., Fesseha, I., Brantly,

E.P., Thomson, M.C.: Malaria stratification, climate, and epidemic early warning in Eritrea. American Journal of Tropical Medicine and Hygiene **77**(6), 61–68 (2007)

[30] Thomson, M.C., Doblas-Reyes, F.J., Mason, S.J., Hagedorn, R., Connor, S.J., Phindela, T., Morse, A.P., Palmer, T.N.: Malaria early warnings based on seasonal climate forecasts from multi-model ensembles. Nature **439**(7076), 576–579 (2006)

[31] Pascual, M., Cazelles, B., Bouma, M.J., Chaves, L.F., Koelle, K.: Shifting patterns: Malaria dynamics and rainfall variability in an African highland. Proceedings of the Royal Society B-Biological Sciences **275**(1631), 123–132 (2008)

[32] Beguin, A., Hales, S., Rocklov, J., Astrom, C., Louis, V.R., Sauerborn, R.: The opposing effects of climate change and socio-economic development on the global distribution of malaria. Global Environmental Change-Human and Policy Dimensions **21**(4), 1209–1214 (2011)

[33] Kulkarni, M.A., Desrochers, R.E., Kerr, J.T.: High Resolution Niche Models of Malaria Vectors in Northern Tanzania: A New Capacity to Predict Malaria Risk? Plos One **5**(2) (2010)

[34] Midekisa, A., Senay, G., Henebry, G.M., Semuniguse, P., Wimberly, M.C.: Remote sensing-based time series models for malaria early warning in the highlands of Ethiopia. Malaria Journal **11** (2012)

[35] Rahman, A., Krakauer, N., Roytman, L., Goldberg, M., Kogan, F.: Application of Advanced Very High Resolution Radiometer (AVHRR)-based Vegetation Health Indices for Estimation of Malaria Cases. American Journal of Tropical Medicine and Hygiene **82**(6), 1004–1009 (2010)

[36] Rahman, A., Kogan, F., Roytman, L., Goldberg, M., Guo, W.: Modelling and prediction of malaria vector distribution in Bangladesh from remote-sensing data. International Journal of Remote Sensing **32**(5), 1233–1251 (2011)

[37] Gemperli, A., Sogoba, N., Fondjo, E., Mabaso, M., Bagayoko, M., Briet, O.J.T., Anderegg, D., Liebe, J., Smith, T., Vounatsou, P.: Mapping malaria transmission in West and Central Africa. Tropical Medicine & International Health **11**(7), 1032–1046 (2006)

[38] Gaudart, J., Toure, O., Dessay, N., Dicko, A.L., Ranque, S., Forest, L., Demongeot, J., Doumbo, O.K.: Modelling malaria incidence with environmental dependency in a locality of Sudanese savannah area, Mali. Malaria Journal **8**, 61 (2009)

[39] Haque, U., Hashizume, M., Glass, G.E., Dewan, A.M., Overgaard, H.J., Yamamoto, T.: The Role of Climate Variability in the Spread of Malaria in Bangladeshi Highlands. Plos One **5**(12) (2010)

[40] Creasey, A., Giha, H., Hamad, A.A., El Hassan, I.M., Theander, T.G., Arnot, D.E.: Eleven years of malaria surveillance in a Sudanese village highlights unexpected variation in individual disease susceptibility and outbreak severity. Parasitology **129**, 263–271 (2004)
