# Supplementary figures and images for: Seasonality of Plasmodium falciparum transmission: a systematic review
Source: Malar J. 2015 Sep 15;14:343. doi: 10.1186/s12936-015-0849-2 (PMC4570512; doi:10.1186/s12936-015-0849-2)

Number of papers

- Simple
- Regression
- Spatial and/or Bayesian
- Mechanistic
- Both
- Other

1980-1985

1990-1995

2000-2005

2010-2013

Time

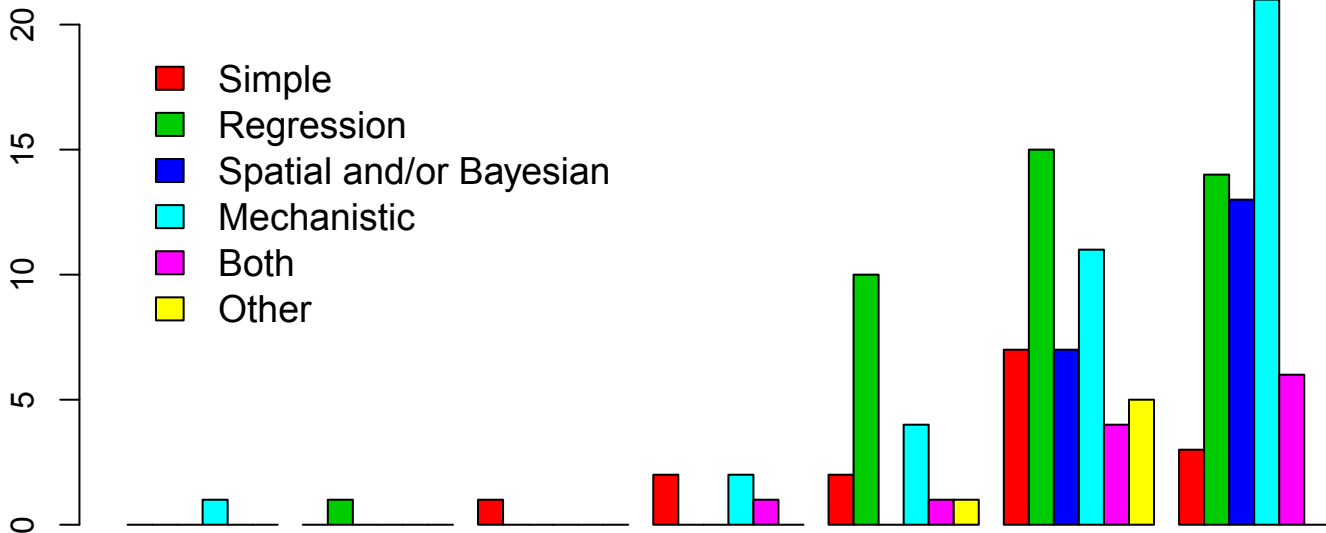

Supplement: Additional file 16: — Number of publications concerning seasonality of malaria by year Temporal trend in the publication of models included in the bibliography, grouped by modeling approach and binned by five-year period. “Both” represents papers that consider both a mechanistic and statistical modeling approach within the same publication. [file 12936_2015_849_MOESM16_ESM.pdf]

## Global distribution of mechanistic models

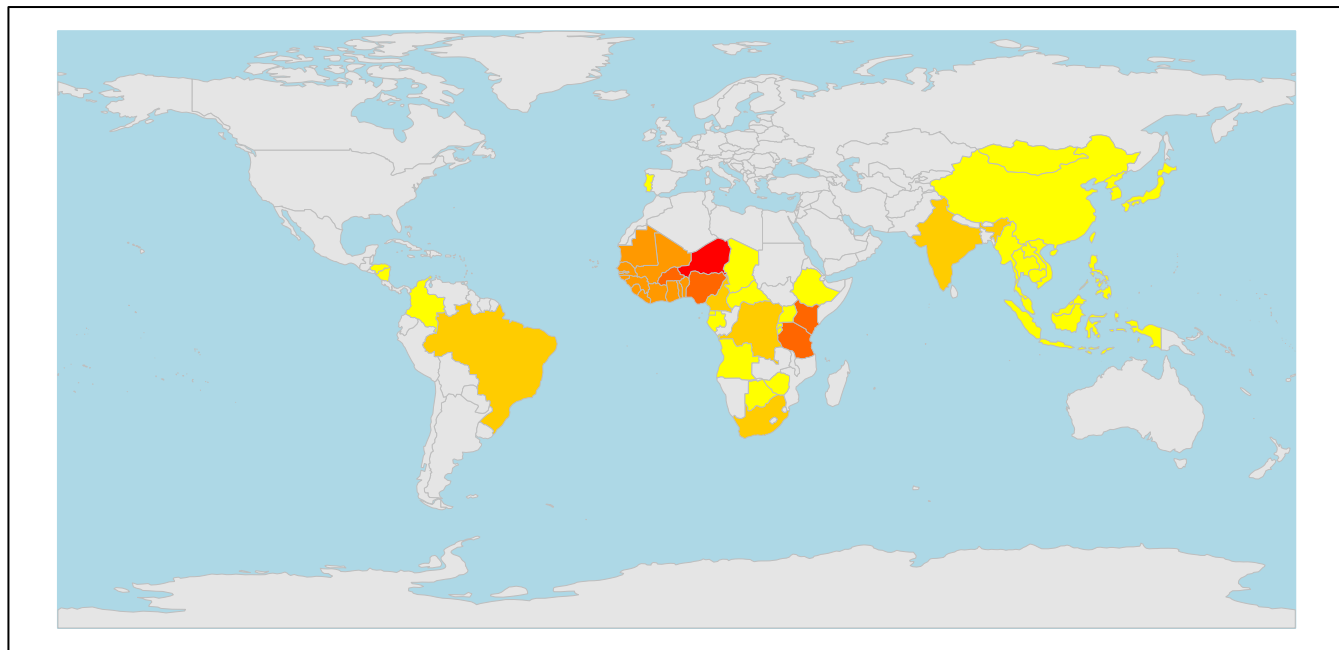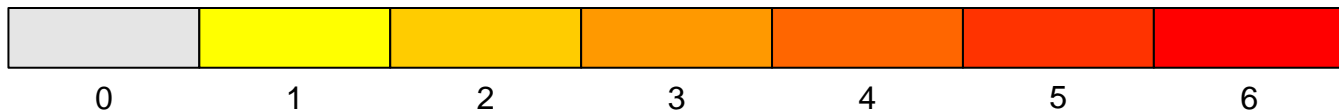

Supplement: Additional file 17: — Global distribution of malaria seasonality papers using mechanistic models. The frequency of the use of mechanistic models for studying malaria seasonality by country is plotted. [file 12936_2015_849_MOESM17_ESM.pdf]
