# Supplementary material for: Seasonality of Plasmodium falciparum transmission: a systematic review
Source: Malar J. 2015 Sep 15;14:343. doi: 10.1186/s12936-015-0849-2 (PMC4570512; doi:10.1186/s12936-015-0849-2)
Supplement: Additional file 18: — Distribution of malaria seasonality papers by malaria metric. The frequency with which different proxies of malaria transmission were studied is plotted for (panel A) mosquito abundance, (panel B) incidence, (panel C) EIR and (panel D) prevalence. [file 12936_2015_849_MOESM18_ESM.pdf]

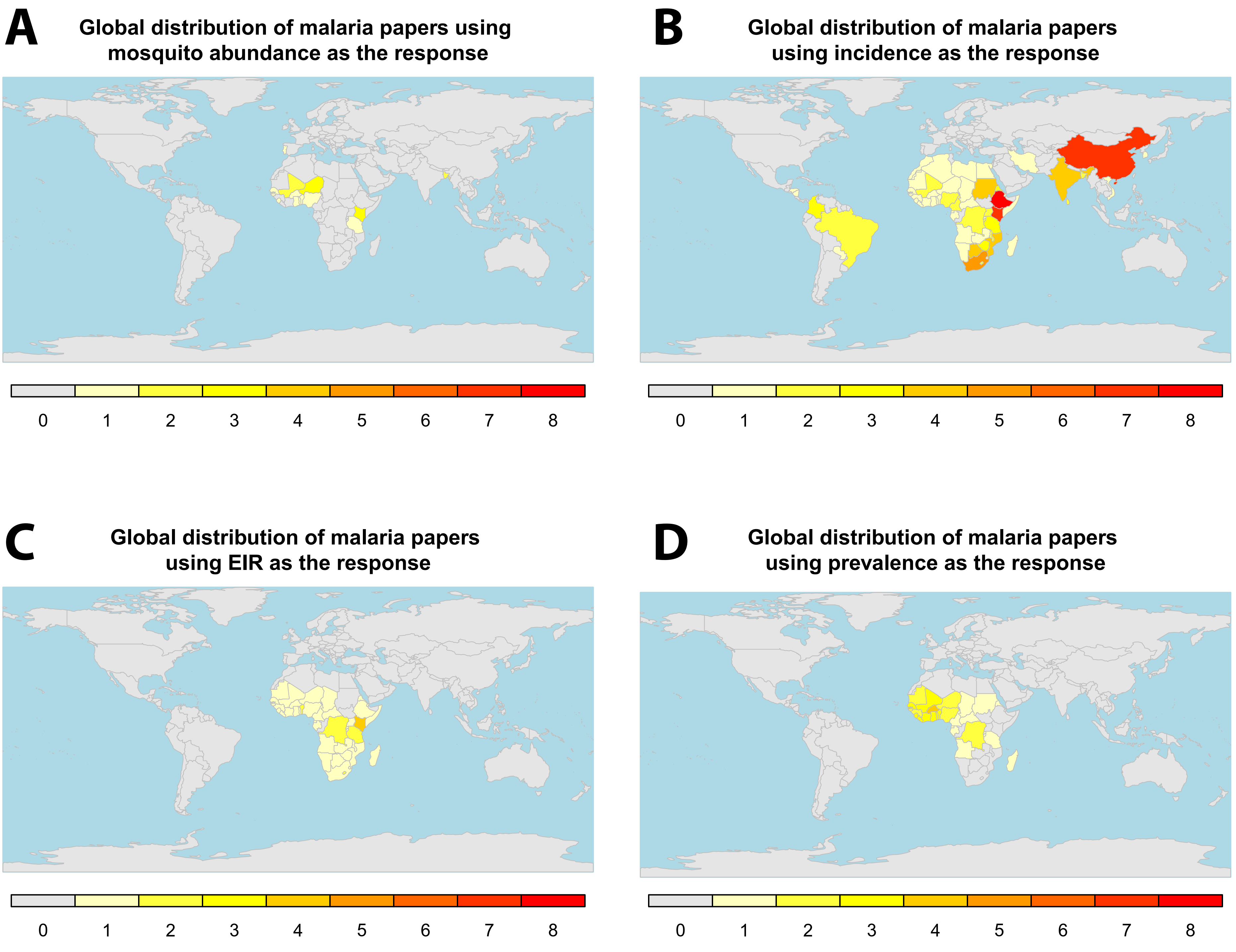
A
Global distribution of malaria papers using mosquito abundance as the response
0 1 2 3 4 5 6 7 8
B
Global distribution of malaria papers using incidence as the response
0 1 2 3 4 5 6 7 8
C
Global distribution of malaria papers using EIR as the response
0 1 2 3 4 5 6 7 8
D
Global distribution of malaria papers using prevalence as the response
0 1 2 3 4 5 6 7 8
