# Supplementary material for: Seasonality of Plasmodium falciparum transmission: a systematic review
Source: Malar J. 2015 Sep 15;14:343. doi: 10.1186/s12936-015-0849-2 (PMC4570512; doi:10.1186/s12936-015-0849-2)
Supplement: Additional file 19: — Systematic review complete list of manuscripts reviewed. [file 12936_2015_849_MOESM19_ESM.pdf]

References

[1] Abdullah, S., Adazu, K., Masanja, H., Diallo, D., Hodgson, A., Ilboudo-Sanogo, E., Nhacolo, A., Owusu-Agyei, S., Thompson, R., Smith, T., Binka, F.N.: Patterns of age-specific mortality in children in endemic areas of sub-saharan Africa. American Journal of Tropical Medicine and Hygiene **77**(6), 99–105 (2007)

[2] Abeku, T.A., De Vlas, S.J., Borsboom, G., Tadege, A., Gebreyesus, Y., Gebreyohannes, H., Alamirew, D., Seifu, A., Nagelkerke, N.J.D., Habbema, J.D.F.: Effects of meteorological factors on epidemic malaria in Ethiopia: a statistical modelling approach based on theoretical reasoning. Parasitology **128**, 585–593 (2004)

[3] Abeku, T.A., van Oortmarssen, G.J., Borsboom, G., de Vlas, S.J., Habbema, J.D.F.: Spatial and temporal variations of malaria epidemic risk in Ethiopia: factors involved and implications. Acta Tropica **87**(3), 331–340 (2003)

[4] Abellana, R., Ascaso, C., Aponte, J., Saute, F., Nhalungo, D., Nhacolo, A., Alonso, P.: Spatio-seasonal modeling of the incidence rate of malaria in Mozambique. Malar J **7**, 228 (2008)

[5] Ageep, T.B., Cox, J., Hassan, M.M., Knols, B.G.J., Benedict, M.Q., Malcolm, C.A., Babiker, A., El Sayed, B.B.: Spatial and temporal distribution of the malaria mosquito *Anopheles arabiensis* in northern Sudan: influence of environmental factors and implications for vector control. Malaria Journal **8** (2009)

[6] Alonso, D., Bouma, M.J., Pascual, M.: Epidemic malaria and warmer temperatures in recent decades in an East African highland. Proceedings of the Royal Society B-Biological Sciences **278**(1712), 1661–1669 (2011)

[7] Amek, N., Bayoh, N., Hamel, M., Lindblade, K.A., Gimnig, J.E., Odhiambo, F., Laserson, K.F., Slutsker, L., Smith, T., Vounatsou, P.: Spatial and temporal dynamics of malaria transmission in rural Western Kenya. Parasites & Vectors **5** (2012)

[8] Aribot, G., Rogier, C., Sarthou, J.L., Trape, J.F., Balde, A.T., Druilhe, P., Roussilhon, C.: Pattern of immunoglobulin isotype response to *Plasmodium falciparum* blood-stage antigens in individuals living in a holoendemic area of Senegal (Dielmo, West Africa). American Journal of Tropical Medicine and Hygiene **54**(5), 449–457 (1996)

[9] Badu, K., Afrane, Y.A., Larbi, J., Stewart, V.A., Waitumbi, J., Angov, E., Ong'echa, J.M., Perkins, D.J., Zhou, G., Githeko, A., Yan, G.: Marked variation in MSP-1(19) antibody responses to malaria in western Kenyan highlands. Bmc Infectious Diseases **12** (2012)

[10] Baeza, A., Bouma, M.J., Dobson, A.P., Dhiman, R., Srivastava, H.C., Pascual, M.: Climate forcing and desert malaria: the effect of irrigation. Malaria Journal **10** (2011)

[11] Balls, M.J., Bodker, R., Thomas, C.J., Kisinza, W., Msangeni, H.A., Lindsay, S.W.: Effect of topography on the risk of malaria infection in the Usambara Mountains, Tanzania. Transactions of the Royal Society of Tropical Medicine and Hygiene **98**(7), 400–408 (2004)

[12] Bantje, H.: Seasonality of births and birth-weights in Tanzania. Social Science & Medicine **24**(9), 733–739 (1987)

[13] Baragatti, M., Fournet, F., Henry, M.-C., Assi, S., Ouedraogo, H., Rogier, C., Salem, G.

[14] Barros, F.S.M., Arruda, M.E., Gurgel, H.C., Honorio, N.A.: Spatial clustering and longitudinal variation of *Anopheles darlingi* (Diptera: Culicidae) larvae in a river of the Amazon: the importance of the forest fringe and of obstructions to flow in frontier malaria. Bulletin of Entomological Research **101**(6), 643–658 (2011)

[15] Bayoh, M.N., Thomas, C.J., Lindsay, S.W.: Mapping distributions of chromosomal forms of *Anopheles gambiae* in West Africa using climate data. Medical and Veterinary Entomology **15**(3), 267–274 (2001)

[16] Becher, H., Kynast-Wolf, G., Sie, A., Ndugwa, R., Ramroth, H., Kouyate, B., Mueller, O.: Patterns of malaria: Cause-specific and all-cause mortality in a malaria-endemic area of West Africa. American Journal of Tropical Medicine and Hygiene **78**(1), 106–113 (2008)

[17] Bi, P., Tong, S.L., Donald, K., Parton, K.A., Ni, J.F.: Climatic variables and transmission of malaria: A 12-year data analysis in Shuchen County, China. Public Health Reports **118**(1), 65–71 (2003)

[18] Bodker, R., Akida, J., Shayo, D., Kisinza, W., Msangeni, H.A., Pedersen, E.M., Lindsay, S.W.: Relationship between altitude and intensity of malaria transmission in the Usambara Mountains, Tanzania. Journal of Medical Entomology **40**(5), 706–717 (2003)

[19] Bomblies, A., Duchemin, J.-B., Eltahir, E.A.B.: A mechanistic approach for accurate simulation of village scale malaria transmission. Malaria Journal **8**, 223 (2009)

[20] Bomblies, A., Duchemin, J.-B., Eltahir, E.A.B.: Hydrology of malaria: Model development and application to a Sahelian village. Water Resources Research **44**(12) (2008)

[21] Briet, O.J.T., Vounatsou, P., Amerasinghe, P.H.: Malaria seasonality and rainfall seasonality in Sri Lanka are correlated in space. Geospatial Health **2**(2), 183–190 (2008)

[22] Briet, O.J.T., Vounatsou, P., Gunawardena, D.M., Galappaththy, G.N.L., Amerasinghe, P.H.: Temporal correlation between malaria and rainfall in Sri Lanka. Malaria Journal **7** (2008)

[23] Camargo, L.M.A., Colletto, G., Ferreira, M.U., Gurgel, S.D., Escobar, A.L., Marques, A., Krieger, H., Camargo, E.P., DaSilva, L.H.P.: Hypoendemic malaria in Rondonia (Brazil, western Amazon region): Seasonal variation and risk groups in an urban locality. American Journal of Tropical Medicine and Hygiene **55**(1), 32–38 (1996)

[24] Ceccato, P., Ghebremeskel, T., Jaiteh, M., Graves, P.M., Levy, M., Ghebreselassie, S., Ogbamariam, A., Barnston, A.G., Bell, M., del Corral, J., Connor, S.J., Fesseha, I., Brantly, E.P., Thomson, M.C.: Malaria stratification, climate, and epidemic early warning in Eritrea. American Journal of Tropical Medicine and Hygiene **77**(6), 61–68 (2007)

[25] Clements, A.C.A., Barnett, A.G., Cheng, Z.W., Snow, R.W., Zhou, H.N.: Space-time variation of malaria incidence in Yunnan province, China. Malaria Journal **8** (2009)

[26] Cottrell, G., Kouwaye, B., Pierrat, C., le Port, A., Bouraima, A., Fonton, N., Hounkonnou, M.N., Massougbodji, A., Corbel, V., Garcia, A.: Modeling the Influence of Local Environmental Factors on Malaria Transmission in Benin and Its Implications for Cohort Study. Plos One **7**(1) (2012)

[27] Craig, M.H., Kleinschmidt, I., Nawn, J.B., Le Sueur, D., Sharp, B.L.: Exploring 30 years of malaria case data in KwaZulu-Natal, South Africa: Part I. The impact of climatic factors. Tropical Medicine & International Health **9**(12), 1247–1257 (2004)

[28] Craig, M.H., Snow, R.W., le Sueur, D.: A climate-based distribution model of malaria transmission in sub-Saharan Africa. Parasitol Today **15**(3), 105–111 (1999)

[29] Creasey, A., Giha, H., Hamad, A.A., El Hassan, I.M., Theander, T.G., Arnot, D.E.: Eleven years of malaria surveillance in a Sudanese village highlights unexpected variation in individual disease susceptibility and outbreak severity. Parasitology **129**, 263–271 (2004)

[30] Devi, N.P., Jauhari, R.K.: Climatic variables and malaria incidence in Dehradun, Uttaranchal, India. Journal of Vector Borne Diseases **43**(1), 21–28 (2006)

[31] Dicko, A., Mantel, C., Kouriba, B., Sagara, I., Thera, M.A., Doumbia, S., Diallo, M., Poudiougou, B., Diakite, M., Doumbo, O.K.: Season, fever prevalence and pyrogenic threshold for malaria disease definition in an endemic area of Mali. Tropical Medicine & International Health **10**(6), 550–556 (2005)

[32] Dimitrov, B.D., Valev, D., Werner, R., Atanassova, P.A.: Cyclic patterns of malaria incidence in Burundi. Central European Journal of Biology **6**(1), 58–67 (2011)

[33] Drakeley, C.J., Carneiro, I., Reyburn, H., Malima, R., Lusingu, J.P.A., Cox, J., Theander, T.G., Nkya, W., Lemnge, M.M., Riley, E.M.: Altitude-dependent and -independent variations in *Plasmodium falciparum* prevalence in northeastern Tanzania. Journal of Infectious Diseases **191**(10), 1589–1598 (2005)

[34] Ermert, V., Fink, A.H., Jones, A.E., Morse, A.P.: Development of a new version of the Liverpool Malaria Model. I. Refining the parameter settings and mathematical formulation of basic processes based on a literature review. Malaria Journal **10** (2011)

[35] Ermert, V., Fink, A.H., Jones, A.E., Morse, A.P.: Development of a new version of the Liverpool Malaria Model. II. Calibration and validation for West Africa. Malaria Journal **10** (2011)

[36] Gao, H.-W., Wang, L.-P., Liang, S., Liu, Y.-X., Tong, S.-L., Wang, J.-J., Li, Y.-P., Wang, X.-F., Yang, H., Ma, J.-Q., Fang, L.-Q., Cao, W.-C.: Change in Rainfall Drives Malaria Re-Emergence in Anhui Province, China. Plos One **7**(8) (2012)

[37] Gaudart, J., Toure, O., Dessay, N., Dicko, A.L., Ranque, S., Forest, L., Demongeot, J., Doumbo, O.K.: Modelling malaria incidence with environmental dependency in a locality of Sudanese savannah area, Mali. Malaria Journal **8**, 61 (2009)

[38] Gemperli, A., Sogoba, N., Fondjo, E., Mabaso, M., Bagayoko, M., Briet, O.J.T., Anderegg, D., Liebe, J., Smith, T., Vounatsou, P.: Mapping malaria transmission in West and Central Africa. Tropical Medicine & International Health **11**(7), 1032–1046 (2006)

[39] Giha, H.A., Rosthoj, S., Dodoo, D., Hviid, L., Satti, G.M.H., Scheike, T., Arnot, D.E., Theander, T.G.: The epidemiology of febrile malaria episodes in an area of unstable and seasonal transmission. Transactions of the Royal Society of Tropical Medicine and Hygiene **94**(6), 645–651 (2000)

[40] Gilioli, G., Mariani, L.: Sensitivity of *Anopheles gambiae* population dynamics to meteo-hydrological variability: a mechanistic approach. Malaria Journal **10** (2011)

[41] Gosoniu, L., Vounatsou, P., Sogoba, N., Maire, N., Smith, T.: Mapping malaria risk in West Africa using a Bayesian nonparametric non-stationary model. Computational Statistics & Data Analysis **53**(9), 3358–3371 (2009)

[42] Goswami, P., Murty, U.S., Mutheneni, S.R., Kukkuthady, A., Krishnan, S.T.: A Model of Malaria Epidemiology Involving Weather, Exposure and Transmission Applied to North East India. Plos One **7**(11) (2012)

[43] Graves, P.M., Osgood, D.E., Thomson, M.C., Sereke, K., Araia, A., Zerom, M., Ceccato, P., Bell, M., del Corral, J., Ghebreselassie, S., Brantly, E.P., Ghebremeskel, T.: Effectiveness of malaria control during changing climate conditions in Eritrea, 1998-2003. Tropical Medicine & International Health **13**(2), 218–228 (2008)

[44] Haghdoost, A.-A., Alexander, N., Cox, J.: Modelling of malaria temporal variations in Iran. Tropical Medicine & International Health **13**(12), 1501–1508 (2008)

[45] Haque, U., Hashizume, M., Glass, G.E., Dewan, A.M., Overgaard, H.J., Yamamoto, T.: The Role of Climate Variability in the Spread of Malaria in Bangladeshi Highlands. Plos One **5**(12) (2010)

[46] Hashizume, M., Chaves, L.F., Minakawa, N.: Indian Ocean Dipole drives malaria resurgence in East African highlands. Scientific Reports **2** (2012)

[47] Hashizume, M., Terao, T., Minakawa, N.: The Indian Ocean Dipole and malaria risk in the highlands of western Kenya. Proceedings of the National Academy of Sciences of the United States of America **106**(6), 1857–1862 (2009)

[48] Hay, S., Renshaw, M., Ochola, S.A., Noor, A.M., Snow, R.W.: Performance of forecasting, warning and detection of malaria epidemics in the highlands of western Kenya. Trends in Parasitology **19**(9), 394–399 (2003)

[49] Huang, F., Zhou, S., Zhang, S., Wang, H., Tang, L.: Temporal correlation analysis between malaria and meteorological factors in Motuo County, Tibet. Malaria Journal **10** (2011)

[50] Ikemoto, T.: Tropical Malaria Does Not Mean Hot Environments. Journal of Medical Entomology **45**(6), 963–969 (2008)

[51] Jackson, M.C., Johansen, L., Furlong, C., Colson, A., Sellers, K.F.: Modelling the effect of climate change on prevalence of malaria in western Africa. Statistica Neerlandica **64**(4), 388–400 (2010)

[52] Jacob, B.G., Muturi, E.J., Mwangangi, J.M., Funes, J., Caamano, E.X., Muriu, S., Shililu, J., Githure, J., Novak, R.J.: Remote and field level quantification of vegetation covariates for malaria mapping in three rice agro-village complexes in Central Kenya. International Journal of Health Geographics **6** (2007)

[53] Jones, A.E., Morse, A.P.: Skill of ENSEMBLES seasonal re-forecasts for malaria prediction in West Africa. Geophysical Research Letters **39** (2012)

[54] Jones, A.E., Morse, A.P.: Application and Validation of a Seasonal Ensemble Prediction System Using a Dynamic Malaria Model. Journal of Climate **23**(15), 4202–4215 (2010)

[55] Jones, A.E., Wort, U.U., Morse, A.P., Hastings, I.M., Gagnon, A.S.: Climate prediction of El Niño malaria epidemics in north-west Tanzania. Malaria Journal **6** (2007)

[56] Kaga, T., Ohta, S.: Ecophysiological and Climatological Effects on Distribution of Vector Species and Malaria Incidence in India. International Journal of Environmental Research and Public Health **9**(12), 4704–4714 (2012)

[57] Kalinga-Chirwa, R., Ngongondo, C., Kalanda-Joshua, M., Kazembe, L., Pemba, D., Kululanga, E.: Linking rainfall and irrigation to clinically reported malaria cases in some villages in Chikhwawa District, Malawi. Physics and Chemistry of the Earth **36**(14-15), 887–894 (2011)

[58] Kazembe, L.N., Chirwa, T.F., Simbeye, J.S., Namangale, J.J.: Applications of Bayesian approach in modelling risk of malaria-related hospital mortality. Bmc Medical Research Methodology **8** (2008)

[59] Kazembe, L.N., Kleinschmidt, I., Sharp, B.L.: Patterns of malaria-related hospital admissions and mortality among Malawian children: an example of spatial modelling of hospital register data. Malaria Journal **5** (2006)

[60] Kiang, R., Adimi, F., Solka, V., Nigro, J., Singhasivanon, P., Sirichaisinthop, J., Leemingsawat, S., Apiwathnasorn, C., Looareesuwan, S.: Meteorological, environmental remote sensing and neural network analysis of the epidemiology of malaria transmission in Thailand. Geospatial Health **1**(1), 71–84 (2006)

[61] Kim, Y.-M., Park, J.-W., Cheong, H.-K.: Estimated Effect of Climatic Variables on the Transmission of Plasmodium vivax Malaria in the Republic of Korea. Environmental Health Perspectives **120**(9), 1314–1319 (2012)

[62] Kitthawee, S., Edman, J.D., Upatham, E.S.: Relationship between female anopheles-dirus (diptera, culicidae) body size and parity in a biting population. Journal of Medical Entomology **29**(6), 921–926 (1992)

[63] Kleinschmidt, I., Sharp, B.L., Clarke, G.P.Y., Curtis, B., Fraser, C.: Use of generalized linear mixed models in the spatial analysis of small-area malaria incidence rates in KwaZulu Natal, South Africa. American Journal of Epidemiology **153**(12), 1213–1221 (2001)

[64] Kulkarni, M.A., Desrochers, R.E., Kerr, J.T.: High Resolution Niche Models of Malaria Vectors in Northern Tanzania: A New Capacity to Predict Malaria Risk? Plos One **5**(2) (2010)

[65] Lafferty, K.D.: The ecology of climate change and infectious diseases. Ecology **90**(4), 888–900 (2009)

[66] Lepers, J.P., Deloron, P., Andriamagatianarason, M.D., Ramanamirija, J.A., Coulanges, P.: Newly transmitted *PLASMODIUM FALCIPARUM* malaria in the central highland plateaus of madagascar - assessment of clinical impact in a rural community. Bulletin of the World Health Organization **68**(2), 217–222 (1990)

[67] Lindsay, S.W., Martens, W.J.: Malaria in the African highlands: past, present and future. Bull World Health Organ **76**(1), 33–45 (1998)

[68] Loha, E., Lindtjorn, B.: Model variations in predicting incidence of Plasmodium falciparum malaria using 1998-2007 morbidity and meteorological data from south Ethiopia. Malaria Journal **9** (2010)

[69] Lou, Y., Zhao, X.-Q.: A climate-based malaria transmission model with structured vector population. Siam Journal on Applied Mathematics **70**(6), 2023–2044 (2010)

[70] Lourenco, P.M., Sousa, C.A., Seixas, J., Lopes, P., Novo, M.T., Almeida, A.P.G.: Anopheles atroparvus density modeling using MODIS NDVI in a former malarious area in Portugal. Journal of Vector Ecology **36**(2), 279–291 (2011)

[71] Mabaso, M.L.H., Craig, M., Vounatsou, P., Smith, T.: Towards empirical description of malaria seasonality in southern Africa: the example of Zimbabwe. Tropical Medicine & International Health **10**(9), 909–918 (2005)

[72] Mabaso, M.L., Craig, M., Ross, A., Smith, T.: Environmental predictors of the seasonality of malaria transmission in Africa: the challenge. Am J Trop Med Hyg **76**(1), 33–38 (2007)

[73] Mala, A.O., Irungu, L.W., Shililu, J.I., Muturi, E.J., Mbogo, C.M., Njagi, J.K., Mukabana, W.R., Githure, J.I.: *Plasmodium falciparum* transmission and aridity: a Kenyan experience from the dry lands of Baringo and its implications for Anopheles arabiensis control. Malaria Journal **10** (2011)

[74] Malone, J.B., Poggi, E., Igualada, F.J., Sintasath, D., Ghebremeskel, T., Corbett, J.D., McCarroll, J.C., Chinnici, P., Shililu, J., McNally, K., Downer, R., Perich, M., Ford, R.: Malaria environmental risk assessment in Eritrea. IGARSS 2003. 2003 IEEE International Geoscience and Remote Sensing Symposium. Proceedings (IEEE Cat. No.03CH37477), 1000–32 (2003)

[75] Manh, B.H., Clements, A.C.A., Thieu, N.Q., Hung, N.M., Hung, L.X., Hay, S.I., Hien, T.T., Wertheim, H.F.L., Snow, R.W., Horby, P.: Social and environmental determinants of malaria in space and time in Viet Nam. International Journal for Parasitology **41**(1), 109–116 (2011)

[76] Mantilla, G., Oliveros, H., Barnston, A.G.: The role of ENSO in understanding changes in Colombia's annual malaria burden by region, 1960-2006. Malaria Journal **8** (2009)

[77] Massad, E., Behrens, R.H., Burattini, M.N., Coutinho, F.A.B.: Modeling the risk of malaria for travelers to areas with stable malaria transmission. Malaria J. **8**, 296 (2009)

[78] Matthys, B., Koudou, B.G., N'Goran, E.K., Vounatsou, P., Gosoniu, L., Kone, M., Cisse, G., Utzinger, J.: Spatial dispersion and characterisation of mosquito breeding habitats in

urban vegetable-production areas of Abidjan, Cote d'Ivoire. Annals of Tropical Medicine and Parasitology **104**(8), 649–666 (2010)

[79] Mbaye, M., Mahe, G., Servat, E., Laganier, R., Bigot, S., Diop, O., Guegan, J.-F.: Water and public health in Sahelian countries: The case of infectious disease in Saint-Louis (Senegal). Secheresse (Montrouge) **20**(1), 161–170 (2009)

[80] Mbogo, C.N.M., Snow, R.W., Khamala, C.P.M., Kabiru, E.W., Ouma, J.H., Githure, J.I., Marsh, K., Beier, J.C.: ELATIONSHIPS BETWEEN PLASMODIUM-FALCIPARUM TRANSMISSION BY VECTOR POPULATIONS AND THE INCIDENCE OF SEVERE DISEASE AT 9 SITES ON THE KENYAN COAST, volume = 52, year = 1995. American Journal of Tropical Medicine and Hygiene (3), 201–206

[81] Midekisa, A., Senay, G., Henebry, G.M., Semuniguse, P., Wimberly, M.C.: Remote sensing-based time series models for malaria early warning in the highlands of Ethiopia. Malaria Journal **11** (2012)

[82] Minakawa, N., Sonye, G., Mogi, M., Githeko, A., Yan, G.Y.: The effects of climatic factors on the distribution and abundance of malaria vectors in Kenya. Journal of Medical Entomology **39**(6), 833–841 (2002)

[83] Mirghani, S.E., Nour, B.Y., Bushra, S.M., Elhassan, I.M., Snow, R.W., Noor, A.M.: The spatial-temporal clustering of *Plasmodium falciparum* infection over eleven years in Gezira State, The Sudan. Malaria Journal **9** (2010)

[84] Moiroux, N., Boussari, O., Djenontin, A., Damien, G., Cottrell, G., Henry, M.-C., Guis, H., Corbel, V.: Dry Season Determinants of Malaria Disease and Net Use in Benin, West Africa. Plos One **7**(1) (2012)

[85] Momota, A., Tabata, K., Futagami, K.: Infectious disease and preventive behavior in an overlapping generations model. Journal of Economic Dynamics & Control **29**(10), 1673–1700 (2005)

[86] Monteiro de Barros, F.S., Honorio, N.A., Arruda, M.E.: Temporal and spatial distribution of malaria within an agricultural settlement of the Brazilian Amazon. Journal of Vector Ecology **36**(1), 159–169 (2011)

[87] Montosi, E., Manzoni, S., Porporato, A., Montanari, A.: An ecohydrological model of malaria outbreaks. Hydrology and Earth System Sciences **16**(8), 2759–2769 (2012)

[88] Musa, M.I., Shohaimi, S., Hashim, N.R., Krishnarajah, I.: A climate distribution model of malaria transmission in Sudan. Geospatial Health **7**(1), 27–36 (2012)

[89] Nkurunziza, H., Gebhardt, A., Pilz, J.: Geo-additive modelling of malaria in Burundi. Malaria Journal **10** (2011)

[90] Nkurunziza, H., Gebhardt, A., Pilz, J.: Bayesian modelling of the effect of climate on malaria in Burundi **9** (2010)

[91] Ohta, S., Kaga, T.: Effect of climate on malarial vector distribution in Monsoon Asia: coupled model for Ecophysiological and Climatological Distribution of mosquito generations (ECD-mg). Climate Research **53**(1), 77–88 (2012)

[92] Owusu-Agyei, S., Dery, D.B., Asante, K.P., Adams, M., Dosoo, D.K., Brown, C., Greenwood, B.: Patterns and seasonality of malaria transmission in a rural endemic area in middle Ghana (Kintampo district). American Journal of Tropical Medicine and Hygiene **73**(6), 155 (2005)

[93] Parham, P.E., Pople, D., Christiansen-Jucht, C., Lindsay, S., Hinsley, W., Michael, E.: Modeling the role of environmental variables on the population dynamics of the malaria vector Anopheles gambiae sensu stricto. Malaria Journal **11** (2012)

[94] Parham, P.E., Michael, E.: Modeling the effects of weather and climate change on malaria transmission. Environmental Health Perspectives **118**(5), 620–626 (2010)

[95] Pascual, M., Cazelles, B., Bouma, M.J., Chaves, L.F., Koelle, K.: Shifting patterns: Malaria dynamics and rainfall variability in an African highland. Proceedings of the Royal Society B-Biological Sciences **275**(1631), 123–132 (2008)

[96] Patz, J.A., Strzepek, K., Lele, S., Hedden, M., Greene, S., Noden, B., Hay, S.I., Kalkstein, L., Beier, J.C.: Predicting key malaria transmission factors, biting and entomological inoculation rates, using modelled soil moisture in Kenya. Tropical Medicine & International Health **3**(10), 818–827 (1998)

[97] Poveda, G., Estrada-Restrepo, O.A., Morales, J.E., Hernandez, O.O., Galeano, A., Osorio, S.: Integrating knowledge and management regarding the climate-malaria linkages in Colombia. Current Opinion in Environmental Sustainability **3**(6), 448–460 (2011)

[98] Rahman, A., Kogan, F., Roytman, L., Goldberg, M., Guo, W.: Modelling and prediction of malaria vector distribution in Bangladesh from remote-sensing data. International Journal of Remote Sensing **32**(5), 1233–1251 (2011)

[99] Rahman, A., Krakauer, N., Roytman, L., Goldberg, M., Kogan, F.: Application of Advanced Very High Resolution Radiometer (AVHRR)-based Vegetation Health Indices for Estimation of Malaria Cases. American Journal of Tropical Medicine and Hygiene **82**(6), 1004–1009 (2010)

[100] Rattanarithikul, R., Konishi, E., Linthicum, K.J.: Detection of *Plasmodium vivax* and *Plasmodium falciparum* circumsporozoite antigen in anopheline mosquitoes collected in southern Thailand. American Journal of Tropical Medicine and Hygiene **54**(2), 114–121 (1996)

[101] Reid, H.L., Haque, U., Roy, S., Islam, N., Clements, A.C.A.: Characterizing the spatial and temporal variation of malaria incidence in Bangladesh, 2007. Malaria Journal **11** (2012)

[102] Roca-Feltrer, A., Carneiro, I., Smith, L., Schellenberg, J.R.M.A., Greenwood, B., Schellenberg, D.: The age patterns of severe malaria syndromes in sub-Saharan Africa across a range of transmission intensities and seasonality settings. Malaria Journal **9** (2010)

[103] Rueda, L.M., Foley, D.H., Peterson, A.T., Wilkerson, R.C.: Geographic and ecologic distribution of the malaria vector, Anopheles sinensis in Korea and other parts of Asia. American Journal of Tropical Medicine and Hygiene **73**(6), 327 (2005)

[104] Ruiz, D., Poveda, G., Velez, I.D., Quinones, M.L., Rua, G.L., Velasquez, L.E., Zuluaga, J.S.: Modelling entomological-climatic interactions of *Plasmodium falciparum* malaria transmission in two Colombian endemic-regions: contributions to a National Malaria Early Warning System. Malaria Journal **5**, 66 (2006)

[105] Ruiz, D., Connor, S.J., Thomson, M.C.: A multimodel framework in support of malaria surveillance and control. In: Thomson, M.C., Beniston, M., GarciaHerrera, R. (eds.) Advances in Global Change Research:HEALTH AND CLIMATE vol. 30, pp. 101–125 (2008)

[106] Singer, B., Cohen, J.E.: Estimating malaria incidence and recovery rates from panel surveys. Mathematical Biosciences **49**(3-4), 273–305 (1980)

[107] Small, J., Goetz, S.J., Hay, S.I.: Climatic suitability for malaria transmission in Africa, 1911-1995. Proceedings of the National Academy of Sciences of the United States of America **100**(26), 15341–15345 (2003)

[108] Snow, R.W., Craig, M.H., Deichmann, U., le Sueur, D.: A preliminary continental risk map for malaria mortality among African children. Parasitology Today **15**(3), 99–104 (1999)

[109] Snow, R.W., Gouws, E., Omumbo, J., Rapuoda, B., Craig, M.H., Tanser, F.C., le Sueur, D., Ouma, J.: Models to predict the intensity of Plasmodium falciparum transmission: applications to the burden of disease in Kenya. Trans R Soc Trop Med Hyg **92**(6), 601–606 (1998)

[110] Sogoba, N., Vounatsou, P., Bagayoko, M.M., Doumbia, S., Dolo, G., Gosoniu, L., Traore, S.F., Toure, Y.T., Smith, T.: The spatial distribution of *Anopheles gambiae sensu stricto* and *An. arabiensis* (Diptera : Culicidae) in Mali. Geospatial Health **1**(2), 213–222 (2007)

[111] Tanser, F.C., Sharp, B., le Sueur, D.: Potential effect of climate change on malaria transmission in Africa. Lancet **362**(9398), 1792–1798 (2003)

[112] Teklehaimanot, H.D., Lipsitch, M., Teklehaimanot, A., Schwartz, J.: Weather-based prediction of Plasmodium falciparum malaria in epidemic-prone regions of Ethiopia I. Patterns of lagged weather effects reflect biological mechanisms. Malar J **3**, 41 (2004)

[113] Teklehaimanot, H.D., Schwartz, J., Teklehaimanot, A., Lipsitch, M.: Weather-based prediction of Plasmodium falciparum malaria in epidemic-prone regions of Ethiopia II. Weather-based prediction systems perform comparably to early detection systems in identifying times for interventions. Malar J **3**, 44 (2004)

[114] Thomson, A.J.: Climate indices, rainfall onset and retreat, and malaria in Nigeria. Journal of Vector Borne Diseases **47**(4), 193–203 (2010)

[115] Thomson, M.C., Connor, S.J.: The development of malaria early warning systems for Africa. Trends in Parasitology **17**(9), 438–445 (2001)

[116] Thomson, M.C., Doblas-Reyes, F.J., Mason, S.J., Hagedorn, R., Connor, S.J., Phindela, T., Morse, A.P., Palmer, T.N.: Malaria early warnings based on seasonal climate forecasts from multi-model ensembles. Nature **439**(7076), 576–579 (2006)

[117] Tian, L., Bi, Y., Ho, S.C., Liu, W., Liang, S., Goggins, W.B., Chan, E.Y.Y., Zhou, S., Sung, J.J.Y.: One-year delayed effect of fog on malaria transmission: a time-series analysis in the rain forest area of Mengla County, south-west China. Malaria Journal **7** (2008)

[118] Tonnang, H.E.Z., Kangalawe, R.Y.M., Yanda, P.Z.: Predicting and mapping malaria under climate change scenarios: the potential redistribution of malaria vectors in Africa. Malaria Journal **9** (2010)

[119] Wayant, N.M., Maldonado, D., Rojas de Arias, A., Cousino, B., Goodin, D.G.: Correlation between normalized difference vegetation index and malaria in a subtropical rain forest undergoing rapid anthropogenic alteration. Geospatial Health **4**(2), 179–190 (2010)

[120] Wimberly, M.C., Midekisa, A., Semuniguse, P., Teka, H., Henebry, G.M., Chuang, T.-W., Senay, G.B.: Spatial synchrony of malaria outbreaks in a highland region of Ethiopia. Tropical Medicine & International Health **17**(10), 1192–1201 (2012)

[121] Worrall, E., Connor, S.J., Thomson, M.C.: A model to simulate the impact of timing, coverage and transmission intensity on the effectiveness of indoor residual spraying (IRS) for malaria control. Tropical Medicine & International Health **12**(1), 75–88 (2007)

[122] Xiao, D., Long, Y., Wang, S., Fang, L., Xu, D., Wang, G., Li, L., Cao, W., Yan, Y.: Spatiotemporal distribution of malaria and the association between its epidemic and climate factors in Hainan, China. Malaria Journal **9** (2010)

[123] Yamana, T.K., Eltahir, E.A.B.: On the use of satellite-based estimates of rainfall temporal distribution to simulate the potential for malaria transmission in rural Africa. Water Resources Research **47** (2011)

[124] Ye, Y., Hoshen, M., Kyobutungi, C., Louis, V.R., Sauerborn, R.: Local scale prediction of Plasmodium falciparum malaria transmission in an endemic region using temperature and rainfall. Global Health Action **2**, 103–115 (2009)

[125] Zacarias, O.P., Andersson, M.: Spatial and temporal patterns of malaria incidence in Mozambique. Malaria Journal **10** (2011)

[126] Zacarias, O.P., Andersson, M.: Mapping malaria incidence distribution that accounts for environmental factors in Maputo Province - Mozambique. Malaria Journal **9** (2010)

[127] Zayeri, F., Salehi, M., Pirhosseini, H.: Geographical mapping and Bayesian spatial modeling incidence in Sistan and Baluchistan province, Iran. Asian Pacific Journal of Tropical Medicine **4**(12), 985–992 (2011)

[128] Zhang, Y., Liu, Q.-Y., Luan, R.-S., Liu, X.-B., Zhou, G.-C., Jiang, J.-Y., Li, H.-S., Li, Z.-F.

[129] Zhang, Y., Bi, P., Hiller, J.E.: Meteorological variables and malaria in a Chinese temperate city: A twenty-year time-series data analysis. Environment International **36**(5), 439–445 (2010)

[130] Zhou, G., Minakawa, N., Githeko, A.K., Yan, G.Y.: Association between climate variability and malaria epidemics in the East African highlands. Proceedings of the National Academy of Sciences of the United States of America **101**(8), 2375–2380 (2004)

[131] Artzy-Randrup, Y., Alonso, D., Pascual, M.: Transmission intensity and drug resistance in malaria population dynamics: implications for climate change. PLoS One **5**(10), 13588 (2010)

[132] Bayoh, M.N., Lindsay, S.W.: Effect of temperature on the development of the aquatic stages of Anopheles gambiae sensu stricto (Diptera : Culicidae). Bulletin of Entomological Research **93**(5), 375–381 (2003)

[133] Beguin, A., Hales, S., Rocklov, J., Astrom, C., Louis, V.R., Sauerborn, R.: The opposing effects of climate change and socio-economic development on the global distribution of malaria. Global Environmental Change-Human and Policy Dimensions **21**(4), 1209–1214 (2011)

[134] Bomblies, A.: Modeling the role of rainfall patterns in seasonal malaria transmission. Climatic Change **112**(3-4), 673–685 (2012)

[135] Childs, D.Z., Boots, M.: The interaction of seasonal forcing and immunity and the resonance dynamics of malaria. Journal of the Royal Society Interface **7**(43), 309–319 (2010)

[136] Chitnis, N., Hardy, D., Smith, T.: A Periodically-Forced Mathematical Model for the Seasonal Dynamics of Malaria in Mosquitoes. Bulletin of Mathematical Biology **74**(5), 1098–1124 (2012)

[137] Dembele, B., Friedman, A., Yakubu, A.-A.: Malaria model with periodic mosquito birth and death rates. Journal of Biological Dynamics **3**(4), 430–445 (2009)

[138] Eckhoff, P.A.: A malaria transmission-directed model of mosquito life cycle and ecology. Malaria Journal **10** (2011)

[139] Edlund, S., Davis, M., Douglas, J.V., Kershenbaum, A., Waraporn, N., Lessler, J., Kaufman, J.H.: A global model of malaria climate sensitivity: comparing malaria response to historic

climate data based on simulation and officially reported malaria incidence. Malaria Journal **11** (2012)

[140] Hoshen, M.B., Morse, A.P.: A weather-driven model of malaria transmission. Malaria Journal **3**, 32 (2004)

[141] Impoinvil, D.E., Cardenas, G.A., Gihture, J.I., Mbogo, C.M., Beier, J.C.: Constant temperature and time period effects on *Anopheles gambiae* egg hatching. Journal of the American Mosquito Control Association **23**(2), 124–130 (2007)

[142] Laneri, K., Bhadra, A., Ionides, E.L., Bouma, M., Dhiman, R.C., Yadav, R.S., Pascual, M.: Forcing Versus Feedback: Epidemic Malaria and Monsoon Rains in Northwest India. Plos Computational Biology **6**(9) (2010)

[143] Mabaso, M.L.H., Kleinschmidt, I., Sharp, B., Smith, T.: El Niño Southern Oscillation (ENSO) and annual malaria incidence in Southern Africa. Transactions of the Royal Society of Tropical Medicine and Hygiene **101**(4), 326–330 (2007)

[144] McKenzie, F.E., Killeen, G.F., Beier, J.C., Bossert, W.H.: Seasonality, parasite diversity, and local extinctions in Plasmodium falciparum malaria. Ecology **82**(10), 2673–2681 (2001)

[145] Morse, A.P., Doblas-Reyes, F.J., Hoshen, M.B., Hagedorn, R., Palmer, T.N.: A forecast quality assessment of an end-to-end probabilistic multi-model seasonal forecast system using a malaria model. Tellus Series a-Dynamic Meteorology and Oceanography **57**(3), 464–475 (2005)

[146] Ngom, R., Siegmund, A.: Urban malaria in Africa: an environmental and socio-economic modelling approach for Yaounde, Cameroon. Natural Hazards **55**(3), 599–619 (2010)

[147] Fisman, D.N.: Seasonality of infectious diseases. In: Annual Review of Public Health vol. 28, pp. 127–143 (2007)

[148] Kelly-Hope, L., Thomson, M.C.: Climate and infectious diseases. In: Thomson, M.C., Beniston, M., GarciaHerrera, R. (eds.) Advances in Global Change Research:HEALTH AND CLIMATE vol. 30, pp. 31–70 (2008)

[149] Grover-Kopec, E.K., Blumenthal, M.B., Ceccato, P., Dinku, T., Omumbo, J.A., Connor, S.J.: Web-based climate information resources for malaria control in Africa. Malaria Journal **5** (2006)

[150] Hoshen, M.B., Morse, A.P.: A Model Structure for Estimating Malaria Risk vol. 9, pp. 41–50 (2005)

[151] Kelly-Hope, L.A., Diggle, P.J., Rowlingson, B.S., Gyapong, J.O., Kyelem, D., Coleman, M., Thomson, M.C., Obsomer, V., Lindsay, S.W., Hemingway, J., Molyneux, D.H.: Short communication: Negative spatial association between lymphatic filariasis and malaria in West Africa. Tropical Medicine & International Health **11**(2), 129–135 (2006)

[152] Lord, C.C.: Seasonal population dynamics and behaviour of insects in models of vector-borne pathogens. Physiological Entomology **29**(3), 214–222 (2004)
